# Optical label-free microscopy characterization of dielectric nanoparticles†

**DOI:** 10.1039/d4nr03860f

**Published:** 2025-02-25

**Authors:** Berenice García Rodríguez, Erik Olsén, Fredrik Skärberg, Giovanni Volpe, Fredrik Höök, Daniel Sundås Midtvedt

**Affiliations:** a Department of Physics, University of Gothenburg Gothenburg Sweden daniel.midtvedt@physics.gu.se; b Department of Physics, Chalmers University of Technology Gothenburg Sweden erik.olsen@msl.ubc.ca

## Abstract

In order to relate nanoparticle properties to function, fast and detailed particle characterization is needed. The ability to characterize nanoparticle samples using optical microscopy techniques has drastically improved over the past few decades; consequently, there are now numerous microscopy methods available for detailed characterization of particles with nanometric size. However, there is currently no “one size fits all” solution to the problem of nanoparticle characterization. Instead, since the available techniques have different detection limits and deliver related but different quantitative information, the measurement and analysis approaches need to be selected and adapted for the sample at hand. In this tutorial, we review the optical theory of single particle scattering and how it relates to the differences and similarities in the quantitative particle information obtained from commonly used label-free microscopy techniques, with an emphasis on nanometric (submicron) sized dielectric particles. Particular emphasis is placed on how the optical signal relates to mass, size, structure, and material properties of the detected particles and to its combination with diffusivity-based particle sizing. We also discuss emerging opportunities in the wake of new technology development, including examples of adaptable python notebooks for deep learning image analysis, with the ambition to guide the choice of measurement strategy based on various challenges related to different types of nanoparticle samples and associated analytical demands.

## Abstract 2

Different optical microscopy methods provide different but related single particle information. This tutorial reviews how optical microscopy images can be related to particle properties, including optical theory and strategies for image analysis.

## Graphical abstract

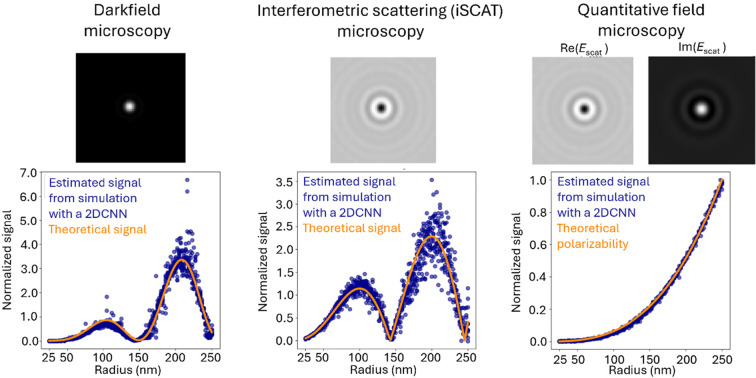

## 1 Introduction

Accurate nanoparticle characterization in terms of size, shape, and composition in complex biological environments is crucial to understanding the relation between nanoparticle structure and function as well as to achieving the full potential of nanoparticle-assisted applications within several fields, including drug delivery^1,2^ and medical diagnostics.^3,4^ Traditionally, such characterization has been performed at the individual particle level using high spatial resolution methods, such as cryogenic transmission electron microscopy (cryo-TEM),^5^ whereas light scattering techniques have been employed to perform quick nanoparticle characterization on an ensemble level. Two examples of such light scattering techniques routinely used to characterize nanoparticle suspensions are dynamic light scattering (DLS),^6^ connecting particle diffusivity to size, and multi-angle light scattering (MALS) to characterize both size and structure.^7,8^

However, none of these approaches are satisfactory for characterizing heterogeneous nanoparticle samples: electron microscopy is an *ex situ* approach suffering from low throughput, while ensemble approaches measure an averaged signal over many individual particles, masking their underlying heterogeneity.^9^ This is particularly problematic for biological nanoparticles, which often display pronounced heterogeneity in terms of size and composition, which may also be a deciding factor for their biological function.^10^

In this context, single-particle characterization using label-free optical microscopy has emerged as an alternative route, achieving widespread use in the last two decades. In fact, the first use of optical microscopy for the characterization of nanoparticles was done more than 100 years ago, which relied on orthogonal illumination and detection pathways to achieve darkfield microscopy.^11^ Although nanoparticles are smaller than the optical resolution limit, it is still possible to detect individual nanoparticles as long as the signal-to-noise is high enough, which in turn enables detailed measurements on the single particle level. Modern implementations typically employ temporally coherent illumination using lasers to image the particles onto sensitive cameras and quantify a combination of the scattering signal and particle motion to achieve high-throughput characterization of particle samples.^12–16^ From the nanoparticle motion, the size is estimated *via* the Stokes–Einstein relation, which relates diffusivity to size for spherical particles in a viscous medium.^17^ The use of nanoparticle motion to estimate size has achieved widespread application under the name nanoparticle tracking analysis (NTA) and exemplifies how scattering microscopy extends traditional ensemble-based characterization approaches to characterize nanoparticles with single nanoparticle resolution.^14,15,18^

Going beyond diffusivity-based particle sizing, over the past decade numerous optical microscopy methods have been developed, aiming at multiparametric nanoparticle characterization with single-particle resolution.^13–16,19–23^ All these techniques are based on the following fundamental observation: the amount of light scattered and absorbed by an object is to a first approximation dependent on its volume and refractive index contrast relative to the surrounding medium. The refractive index is a complex-valued material-specific property that dictates the efficiency of a material to scatter and absorb light.^24^ The real part of the refractive index governs light scattering, while the imaginary part of the refractive index governs light absorption. Thus, optical nanoparticle characterization can distinguish between different types of particles based on their ability to scatter and absorb light.^12,25^ In the specific case of biological nanoparticles, particle refractive index relates to particle dry mass density and the measured optical particle signal is to a first approximation either proportional to or quadratically related to particle dry mass, where the exact relation depends on the used imaging technique.^26–28^ In addition to the scattering amplitude, the relative amount of scattering to different scattering angles can also be used to characterize nanoparticle samples.^23,29^

**Fig. 1:**
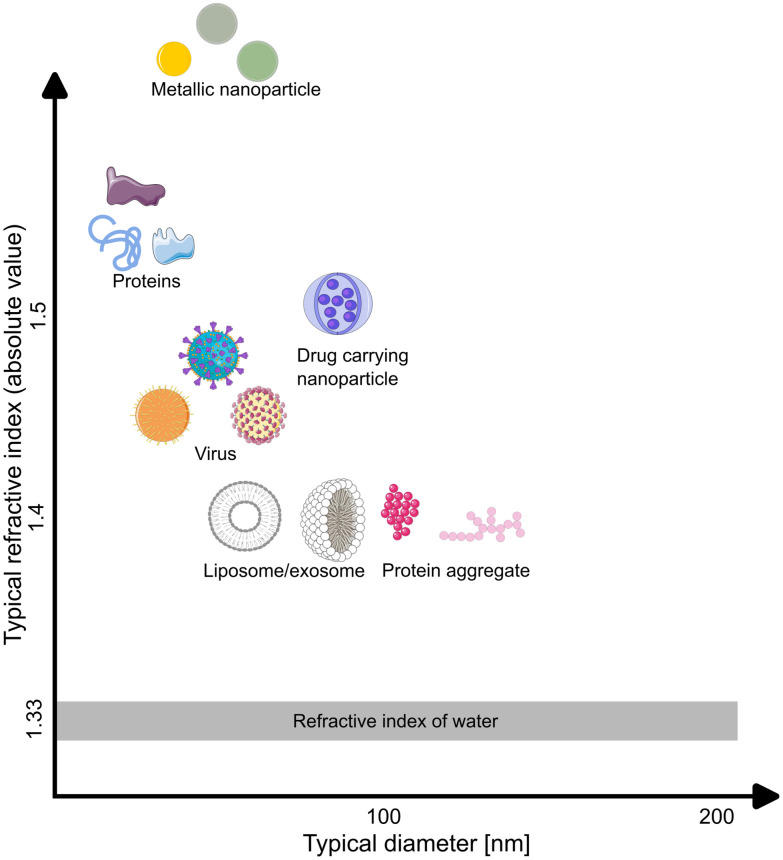
Typical nanoparticles studied using elastic scattering microscopy, organized by size and refractive index. The size range of nanoparticles typically studied by such microscopy techniques ranges from individual biomolecules to large biomolecular complexes, where specific size and refractive index ranges are shown in Table 1.

To give some specific examples, some types of nanoparticles that can be characterized using elastic scattering microscopy are highlighted in Fig. 1, where typical values of the respective sizes and refractive indices are shown in Table 1. For objects much smaller than the wavelength of the illuminating light, such as individual biomolecules, the relative scattering amplitude at different angles has a weak particle size dependence.^7,8^ For such particles, it is sufficient to determine the scattering amplitude in a limited range of angles to characterize the particle mass. This has been utilized to determine the mass of individual proteins.^12,27,42^ For larger biomolecular complexes, such as viruses, liposomes, and protein aggregates, the scattered light amplitude integrated over all scattering angles is still related to their mass. However, interference effects between the scattered light from individual molecular elements within the complexes generate a directionality of the scattered light such that the light amplitude measured in a elastic scattering microscope will depend on the measurement geometry.^24^ For instance, a liposome, consisting of a lipid bilayer shell with a water-filled core, will scatter light differently from a drug-containing nanoparticle of the same size and mass, simply due to a different spatial distribution of biomolecules. This fact has been used to distinguish empty liposomes from exosomes filled with biological material through simultaneous characterization of scattering amplitude and size.^15^

**Table 1:** Typical values of refractive index and diameter of nanoparticles studied using elastic scattering microscopy techniques

Type of particle	Typical refractive index	Typical diameter
Proteins	1.56–1.61, ref. 27	1 nm–10 nm, ref. 30 and 31
Virus	1.37–1.50, ref. 32 and 33	20 nm–300 nm, ref. 21 and 34
Drug carrying nanoparticle	1.46–1.50, ref. 35	10 nm–1000 nm, ref. 36
Protein aggregate	1.34–1.40, ref. 23 and 37	100 nm–up to microns, ref. 37 and 38
Liposome/exosome	1.35–1.45, ref. 15 and 39	50 nm–1000 nm, ref. 36
Metallic nanoparticle	2–5, ref. 21	1 nm–100 nm, ref. 40

Metallic nanoparticles present another example of widely used nanoparticles that can be characterized using elastic scattering microscopy.^12^ Such nanoparticles interact much more strongly with the illuminating light compared to biological nanoparticles of the same particle size due to the refractive index difference between gold and water being larger than that of biomolecules and water (Fig. 1). Moreover, in contrast to biological nanoparticles, they typically display considerable light absorption in addition to light scattering, as a result of plasmonic resonance. This plasmonic resonance, in turn, depends on particle size and shape.^43^ Thus, to fully characterize such nanoparticles it is important to use an experimental design capable of quantifying both the scattered as well as the absorbed light.

From the above considerations, it becomes clear that while individual biomolecules can be characterized in terms of their dry mass based on the measured signal amplitude, the characterization of larger particles in terms of size, refractive index, and structure requires an experimental design and data analysis pipeline that is optimized for the specifics of the sample. Therefore, although method development and refinement are likely to continue and further expand the information that can be extracted from microscopy images, it will always be imperative to choose an experimental design that maximizes the amount of useful information about the investigated sample in the recorded scattering pattern, and an image analysis approach that optimally utilizes that information.

This tutorial is written with the intention to provide an overview of the label-free particle information contained in microscopy images to guide academic and industrial practitioners entering the field, where the main text contains the core equations to understand the measured microscopy signal and the boxes are there to provide a deeper mathematical background. To guide the reader in this process, section 2 presents a theoretical understanding of how the image is formed in a light scattering microscope, section 3 provides an understanding of how to quantify physical properties of the measured nanoparticles from their corresponding microscopy image, section 4 presents clear guidelines on how to choose the optimal measurement modality for specific purposes, and section 5 provides a toolbox for performing optical nanoparticle characterization using elastic scattering microscopy, in the form of Python notebooks containing ready-to-use code for particle detection and characterization. The importance of considering these aspects is highlighted by a few examples from the literature, where different optical microscopy methods are here divided into three different categories based on their different particle information in the image (Box 1).

**Box 1 Definition of the microscopy categories used in this work**

In this tutorial, **elastic scattering microscopy techniques** refers to all light microscopy methods that can measure individual sub-micron particles without relying on light wavelength shifts from the particle interaction, therefore excluding inelastic methods such as Raman or fluorescence microscopy. The angle between the incoming and outgoing light at the sample can be arbitrary here, thus including transmission, backscattering, and side-scattering methods. Moreover, it is assumed that the light source has a temporal coherence length that is much longer than the size of the nanoparticle, which holds for both LED and laser illumination.^44^ Elastic scattering microscopy methods are further divided into three categories based on the information content in the recorded images.

**Darkfield microscopies** here refers to all microscopy techniques for which the background signal does not reach the camera.

**Interferometric scattering microscopies** here refer to all microscopy techniques where the background signal is non-zero, and the measured signal is the amplitude of the interference signal between the particle and the background signal. Thus, it ranges from brightfield methods such as coherent brightfield microscopy (COBRI) and in-line holography to backscattering methods such as interferometric scattering (iSCAT) microscopy.

**Holographic microscopies** here refers to all microscopy techniques where the information from one or more images is combined with optical imaging theory to obtain the complex-valued optical field¶¶In optical microscopy, the optical (or light) field is often used instead of the electric field. The difference between the two fields is a normalization constant 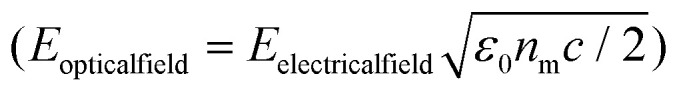 that affects how the fields relate to the measured light intensity, where the measured light intensity at a camera is equal to the squared modulus of the optical field. rather than only the signal amplitude as for the other two categories. This is often done using a modulation of the optical signal, where this modulation can be caused by, for example, an external reference signal or passing the optical signal through a diffraction grating.^45^ This category includes both transmission and non-transmission implementations of methods such as off-axis holography and quadriwave lateral shearing interferometry (QLSI), but also quantitative phase microscopy methods for which the full optical field can in principle be quantified but most often only the phase information is used during the analysis.

## 2 Image formation in a elastic scattering microscope

**Fig. 2:**
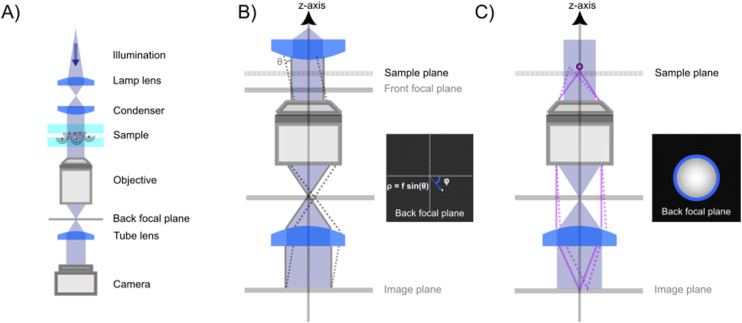
Propagation of the scattered light through a elastic scattering microscope. (A) A general elastic scattering microscopy setup, here depicted in the case of forward scattering. The sample is illuminated by a plane wave. Both the illuminating light and the scattered light from particles in the sample propagate through the optical system and are recorded by a camera. (B) The image obtained by a elastic scattering microscope is largely determined by the objective lens. Plane waves entering into the objective are transformed into a focused spot at the back focal plane. When the objective is illuminated by a plane wave impinging on the objective at an angle *θ* with respect to the optical axis and at an angle *ϕ* relative to the horizontal axis, this tightly focused spot is offset a distance *ρ* = *f* sin *θ* away from the optical axis. (C) The scattered field from a nanoparticle can be considered a linear combination of many plane waves, incident on the objective with different angles *θ* and *ϕ*. Each such plane wave produces a similar spot as described in (B). Summing up, all these plane wave contributions produce a field at the back focal plane as shown in the inset, which, in the case of particles much smaller than the wavelength of light to a first approximation, reassembles that of a plane wave. Notice the sharp cutoff, highlighted as a circle in the inset. This is due to the limited angular range admitted by the objective and is set by the objective NA.

A elastic scattering microscope is a device in which the sample is illuminated by temporally coherent light (Box 1), and the light scattered from the sample is collected and recorded by, for example, a camera or a photomultiplier tube (Fig. 2A). Most commonly, a microscope objective is used to collect the scattered light from the objects (although lens-free solutions exist as well^46^). On the opposite side of the objective, another lens, called the tube lens, collects the light from the objective to form an image onto the camera. The objective, tube lens, and camera together define the *optical axis*.

The properties of the objective and the sample illumination largely determine the scattering information that propagates to the camera in a light microscope. First, consider an objective illuminated by a plane wave, which corresponds to a collimated sample illumination with a constant intensity profile propagating along the optical axis. At the back focal plane of the objective, this wave is focused to a spot centered on the optical axis (Fig. 2B). A plane wave that is tilted by an angle *θ* relative to the optical axis will produce a focused spot at the back focal plane that is offset by a distance *f* sin *θ* relative to the optical axis, where *f* is the back focal length of the objective (Box 2).

**Box 2 Focusing of a plane wave by an objective**

A microscope objective is a lens of finite spatial extent. Since the sample of interest in a microscopy measurement is located close to the working distance of the objective, within the field of view of the microscope, not all light in the sample plane is necessarily captured by the objective. From a mathematical standpoint, the incoming light transmitted through the microscope objective can therefore be represented by a pupil function *P*(*θ*), defining the transmittance of plane waves with incident angle *θ* on the objective (Fig. 2C). For an ideal objective, the pupil function can be written as1
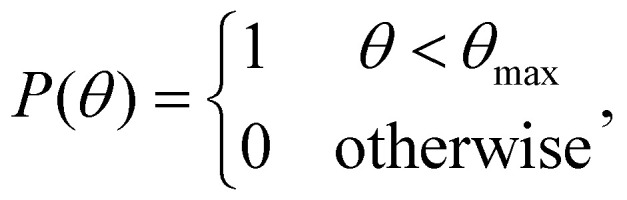
where *θ*_max_ is the largest incident angle accepted by the objective. This is related to the numerical aperture (NA) of the objective as NA = *n*_NA_ sin *θ*_max_, where *n*_NA_ is the refractive index of the media between the objective front lens and the sample.

Now, consider an objective illuminated by a plane wave propagating at an angle *θ* relative to the optical axis and at an angle *ϕ* relative to the *x*-axis. The optical field at the back focal plane is then given by

2 *E*_bfp_(*x*,*y*) = *AP̂*(*k*(*x* − *f* sin *θ* cos *ϕ*),*k*(*y* − *f* sin *θ* sin *ϕ*)),

where *A* is complex-valued and contains the amplitude and phase of the plane wave, the function *P̂* is the transfer function of the objective, *z* = *f* is the back-focal length of the objective lens, and *k* = 2π/*λ* where *λ* is the wavelength of light (Fig. 2B). For an objective without optical aberrations, the transfer function under the paraxial approximation is given by ref. 473
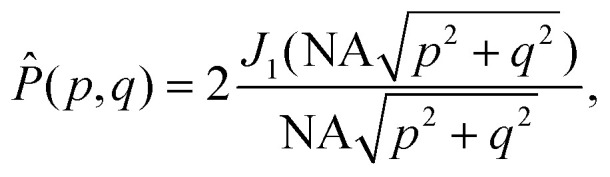
where *J*_1_ is the first-order Bessel function and (*p*,*q*) are spatial frequency values. Thus, all incoming plane waves are transformed into tightly focused spots in the back-focal plane, and the position of each spot in the back-focal plane depends on its incident angle according to eqn (2).

As it turns out, a general incident optical field can be described as a linear combination of plane waves with different incident angles *θ* relative to the optical axis (Box 3), and *ϕ* relative to an at this point arbitrarily defined *x*-axis perpendicular to the optical axis.^48^ Connecting this to the specific topic of this tutorial, consider a nanoparticle located at the front focal plane of the objective (Fig. 2C), illuminated by a plane wave propagating along the optical axis. This produces a scattered optical field *E*, which can be decomposed as a sum of plane waves *Ê*(*θ*,*ϕ*), each propagating with specific angles *θ* and *ϕ*. The field at the back focal plane can then be obtained by summing the contributions from all such plane-wave components, each of which produces a focused spot as in Fig. 2B. Although the general expression is fairly complicated, an approximate form of the field at the back-focal plane can be obtained by utilizing that each plane wave component contributes to the summation only close to the center of the corresponding focused spot. Under this approximation, the field at the back focal plane is given by

4 *E*_bfp_(*ρ* = *f*sin *θ*) ≈ *Ê*(*θ*,*ϕ*) *θ* < *θ*_max_.

**Box 3 Decomposition of the optical field in plane wave components**

Similar to how a time-dependent signal can be decomposed to a sum of contributions with different amplitudes and frequencies, an optical field in a homogeneous environment can generally be decomposed to a sum of plane waves with different wave vectors (or spatial frequencies). Each such plane wave component can mathematically be written as

5 *Φ*_**k**_(**x**) = *e*^*i***k**·**x**^.

Consider a monochromatic optical field *E* (meaning that the field consists of light with a single frequency), evaluated on a plane (the *xy*-plane) perpendicular to the optical axis. Its plane wave decomposition reads6
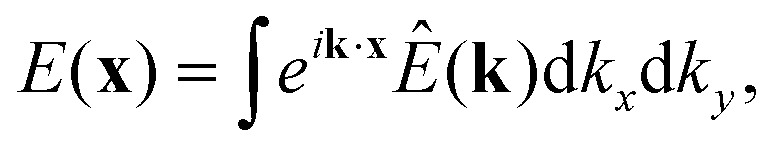
where *Ê*(**k**) is a coefficient describing both amplitude and phase of the plane wave component *Φ*. These components are found through the inverse transform of eqn (6),7
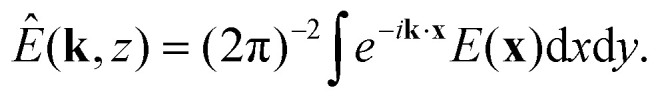

Note that since *k*^2^ = *k*_*x*_^2^ + *k*_*y*_^2^ + *k*_*z*_^2^ = (2π*n*_m_/*λ*), 
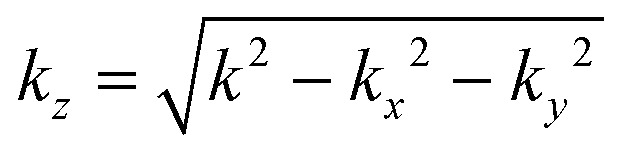
. Therefore, all components with *k*_*x*_^2^ + *k*_*y*_^2^ > *k*^2^ are exponentially decaying in amplitude during propagation in the *z* direction, causing that only components with *k*_*x*_^2^ + *k*_*y*_^2^ ≤ *k*^2^ will be present after long propagation distances, such as propagation through a microscope.

The mathematically versed reader may recognize that these equations resemble Fourier transforms. Denoting the Fourier transform operator by 
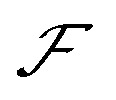
, the above equations can be written as8
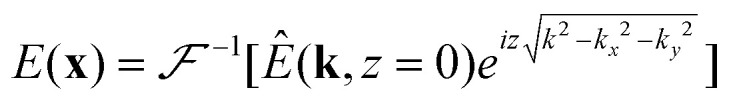
9
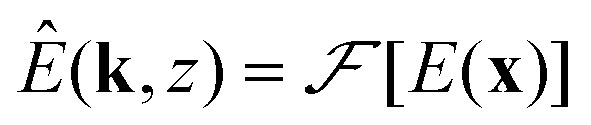
10
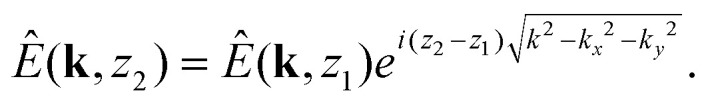

At the camera plane, since only non-decaying scattering components can reach the camera, these expressions are more conveniently expressed in polar coordinates. A optical field at the camera plane with dependence on both the scattering angle *θ* as well as the azimuthal angle *ϕ* can be described by11
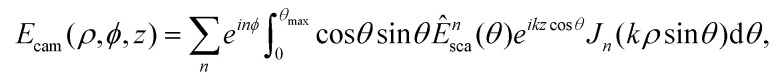
where *J*_*n*_ is the *n*th order Bessel function, *n* are integers, *θ*_max_ comes from the properties of the microscope objective, and the integration runs over the propagation angle *θ* with respect to the optical axis, instead of the wave vector projection **k**. Similarly, the plane wave decomposition can be obtained from the field at the camera plane as12
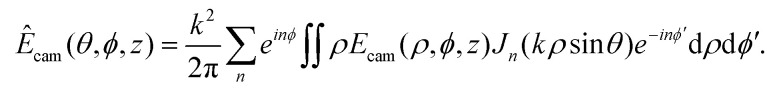

Thus, the field at the back focal plane mirrors the angular distribution of the scattered light, for scattering angles *θ* < *θ*_max_ (Fig. 2C). Note that here the polarization of light is dropped for simplicity. For diffraction of light, scalar diffraction theory, which ignores polarization effects, can often be accurately applied.^49^ However, in some cases, ignoring polarization effects can lead to significant errors. For example, this is evident in the scattering of non-isotropic particles, as described by the equations in section 3.

To form an image of the sample on the camera, a lens (tube lens) is placed between the objective and camera, such that an incoming plane wave to the objective enters the camera as a plane wave, and light scattered from a point source at the focal plane of the objective forms a focused image on the camera. As a result of this, the plane wave components of the field at the camera plane reproduce exactly the plane wave components impinging on the objective, for all plane waves with *θ* < *θ*_max_. Mathematically, this is expressed as

13 *Ê*_cam_(*θ*,*ϕ*) = *Ê*(*θ*,*ϕ*)*P*(*θ*).

Note that in these expressions, a unitary magnification has been implicitly assumed. The effect of magnification will be to spatially scale the recorded image without affecting the range of scattering angles from the particle that reach the camera,^50^ where it is the range of recorded scattering angles that affects the integrated particle signal. Magnification can therefore be taken into account by using the effective pixel size of the camera in the image analysis. Thus, for brevity and unless otherwise stated, we always consider a unitary magnification in this work.

Now, we have a mathematical framework for relating the field at the focal plane to the fields at the back-focal plane and the camera. This framework involves decomposing the field into its plane wave components, applying the optical transfer function, and summing up the contributions from the individual components.

In the context of elastic scattering microscopes, the optical field at the focal plane is typically a superposition of the illuminating field *E*_ill_, and the field scattered by objects in the sample *E*_sca_. The field at the camera is then given by

14 *Ê*_cam_(*θ*,*ϕ*) = *Ê*_ill_(*θ*)*P*(*θ*) + *Ê*_sca_(*θ*,*ϕ*)*P*(*θ*).

However, the microscope camera does not record the angular components of the incident field; instead, it records the spatial distribution of the incident field intensity. This is given by the modulus squared of the spatial distribution of the optical field. The intensity recorded by the camera can be written as15
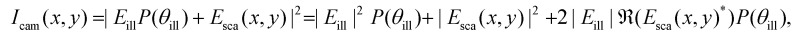
where the spatial distributions of the illuminated and scattered fields are related to their plane wave components through eqn (11) (Box 3). For completeness, in Box 4 the contributions from the individual plane wave components to the measured intensity is described.

**Box 4 Decomposition of the recorded intensity into plane wave components**

The formalism introduced in Boxs 2 and 3 allows us now to investigate how the plane wave components of the scattered and illuminating light contribute to the recorded intensity. At the camera plane, the scattered field is given by the Fourier transform of the field at the back focal plane of the objective. Since the objective only admits plane wave components with angles relative to the optical axis *θ* < *θ*_max_, it follows that it only admits plane waves with projected wave vectors |**k**| ≤ *k* sin *θ*_max_. The scattered field at the camera plane is therefore given by 16
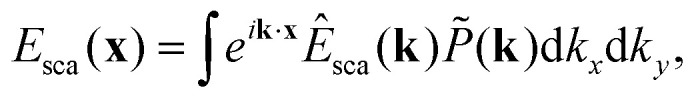
where *P̃* is the pupil function expressed in terms of the projected wave vectors instead of the propagation angle, given by *P̃*(**k**) = 1 for |**k**| ≤ *k* sin *θ*_max_ and 0 otherwise.

In polar coordinates, one has 17
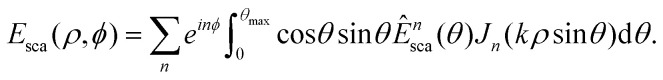

Thus, one finds for the second and last terms in eqn (15)18
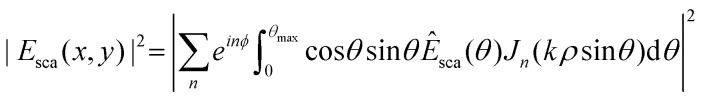
19
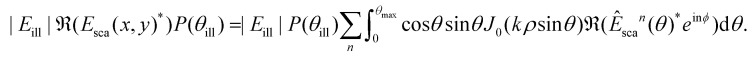

In this expression, it is assumed that the field that reaches the camera is not magnified by the objective. This effect can be taken into account by replacing the argument of *J*_*n*_ with *Mkρ* sin *θ*, where *M* is the magnification.

The first two terms of eqn (15) describe the intensities of the illuminating field and the scattered field independently. The third term describes the interference of the scattered field with the illuminating field and contains information about the relative phases of the two fields. Notice that the first and last of these terms are relevant only for illumination angles for which *P*(*θ*_ill_) > 0.

The amount of light scattered from a nanoparticle is generally much smaller than the amplitude of the light that is incident on it. Thus, in a transmission scattering measurement, as depicted in Fig. 2A, the first term of eqn (15) will dominate over the last two terms, resulting in a small signal-to-background ratio. Three strategies are traditionally used to overcome this signal-to-background ratio limitation for quantitative characterization of subwavelength-sized particles (Box 1); (i) darkfield microscopy enhances the contrast by only allowing the scattered light to reach the camera, (ii) interferometric scattering techniques instead focus on quantifying the interferometric term, while (iii) holographic microscopy uses the interference between different optical fields at the camera plane to obtain the complex-valued optical field of the light that have interacted with the sample (Box 1). All mentioned strategies have in common that they are designed to manipulate one or more of the terms in eqn (15) to enhance their performance, and the details of how this is achieved using the three techniques mentioned above are discussed in the following subsections.

### 2.1 Darkfield microscopies

**Fig. 3:**
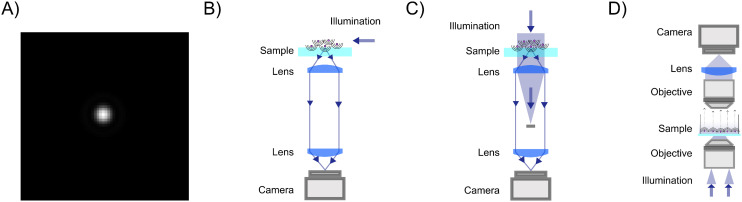
Darkfield microscopy setups: (A) in darkfield microscopy, the particles are visible on the camera as bright spots against a dark background. (B) Darkfield through oblique illumination: the sample is illuminated by light at an angle larger than the maximum angle admitted by the objective so that the illuminating light is prevented from propagating through the optical system. (C) Darkfield using spatial blocking: the illumination light is blocked at the back focal plane using a physical filter. (D) Evanescent field imaging: one utilizes total internal reflection at the interface between glass and sample to produce an evanescent field. This evanescent field amplitude decays exponentially away from the surface and thus will not reach the camera while scattering from particles close to the interface can be recorded on a camera.

Darkfield microscopy is a widely used technique to enhance the contrast of small particles, being a relatively simple but powerful configuration. It has been used for over 100 years^11^ and is still one of the standard techniques to characterize the hydrodynamical radius of nanoparticles using NTA.^13,51^ Darkfield techniques aim to only allow the scattered light to reach the camera, thereby suppressing all terms except the second term in eqn (15). In this way, the scattered light from the particles appears as bright dots against a dark background (Fig. 3A) (hence the name “darkfield”). Since there is no background signal, the darkfield signal is given by

20 *I*_DF_ = |*E*_sca_|^2^.

Such background suppression can be achieved by a vast range of microscope configurations, where the choice of configuration affects the relation between the properties of the particles and the measured signal. The most common ways of achieving such background rejection rely on using an illumination angle outside the range captured by the objective (Fig. 3B), spatial blocking of the excitation beam (Fig. 3C), or an evanescent illumination (Fig. 3D).^12^

Using an illumination angle that lies outside the range captured by the objective achieves full background suppression while still allowing for the particle scattering to be measured for a wide range of different suspended particles (Fig. 3B).^11,52,53^ This approach is commonly used in NTA setups, in which suspended nanoparticles are tracked and characterized based on their Brownian motion.^52^ However, the largest angle admitted by the objective must be smaller than the illumination angle for the incoming light not to be collected, which puts a limit on the plane wave components reaching the camera and, therefore, the information content of the microscope images.

Darkfield microscopy can also be achieved through spatial blocking of the excitation beam using an illumination angle within the numerical aperture of the objective (Fig. 3C). It relies on the observation that at the back focal plane of the objective, the illuminating plane wave is tightly focused, while the scattered field from a point source propagates as a plane wave (Fig. 2).^48^ As a result of this, the excitation beam can be blocked from reaching the camera by placing a small non-transmitting filter at the back focal plane.^54,55^ Specifically, the effect of the filter on the scattered field from the particles can be neglected as long as the physical size of the filter is much smaller than the extent of the scattered field at the back focal plane. This type of darkfield microscope is sensitive to stray light that is not blocked by the optical filter, which limits the background rejection.

Finally, darkfield microscopy can be achieved by using an evanescent field to illuminate the sample (Fig. 3D). An evanescent field is an optical near-field that is present at the interface between two media when the incident light undergoes total internal reflection at the interface. This is the principle of operation of total internal reflection microscopy and waveguide microscopy.^56–58^ The evanescent field is present only very close to the interface (within a fraction of the wavelength of the illuminating light) and does not propagate to the camera. The light that is scattered from the evanescent field, however, does propagate and can therefore be imaged. The evanescent field, therefore, limits the imaging to particles close to an interface.

### 2.2 Interferometric scattering microscopies

A drawback of the darkfield microscopy techniques discussed in the previous section is that the illuminating light is not recorded. Since the scattered field from a nanoparticle is proportional to the illuminating field (see section 4 for details), quantitative nanoparticle characterization using darkfield techniques requires either detailed calibration or separate quantification of the light intensity at the sample plane.

**Fig. 4:**
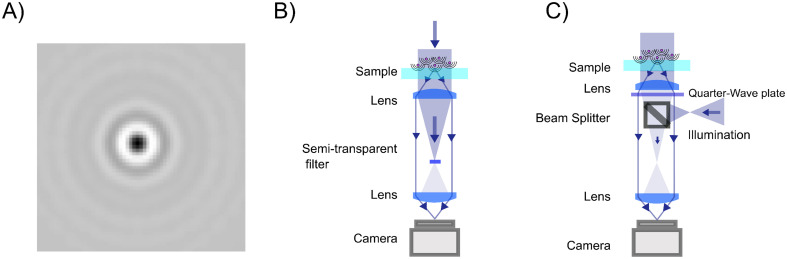
Interferometric scattering techniques setups: (A) in interferometric scattering microscopy, the particle scattering patterns in the microscopy images are often seen as dark spots against a bright background,^27,60^ where both the sign and contrast of the signal depends on the relative phase difference between the particle signal and the background signal (Fig. 8A). (B) Coherent Brightfield microscopy (COBRI): the scattered light is recorded in transmission, and the relative strength of the illumination light and the scattered light reaching the camera is tuned using a semi-transparent filter. (C) Interferometric scattering microscopy (iSCAT): the sample is illuminated from below. Light is partially reflected at the interface between the coverslip and the sample. This partially reflected light is propagated through the optical system and is recorded on the camera. Most of the light is, however, not reflected at the interface and instead produces scattering from nanoparticles in the sample. The scattered light from the nanoparticles interferes with the reflected light to produce interferometric scattering patterns on the camera.

Interferometric microscopy is an approach that circumvents this problem by focusing on quantifying the last term of eqn (15), explicitly containing the illuminating field *E*_ill_. The signal is the interference between the particle signal and a background signal, and therefore, the background signal is non-zero, representing the illuminating field amplitude (Fig. 4A). This ensures an internal reference to which the scattered field can be compared.

However, given the limited dynamic range and read noise of the detector recording the image, the combination of signal-to-noise ratio and signal-to-background ratio will limit the ability to detect weakly scattering particles in this measurement strategy since the particle contrast can not be enhanced by increasing the illuminating light intensity. One way to allow for a stronger illumination, which will increase the particle signal, without saturating the camera is by introducing a partially transmissive filter centered at the back focal plane of the objective (Fig. 4B).^59–62^ Placing optical components in the back focal plane of the objective to improve the measured optical signal goes all the way back to the development of phase contrast.^63^ In the case of a partially transmissive filter centered at the back focal plane of the objective, the presence of the filter can be represented by a function *T*(*θ*),21
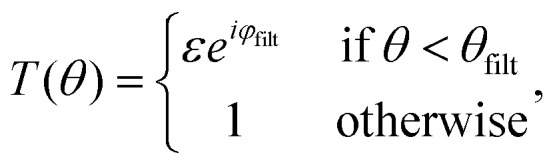
with *ε* < 1, such that the filter attenuates and phase shifts plane waves with incident angles *θ* < *θ*_filt_.^23,59,64^ If *θ*_filt_ ≪ *θ*_max_ andtioned in the back focal plane such that it attenuates the illuminating light before it can reach the camera, the filter will attenuate the illuminating light while leaving the scattered light unaffected in the case of nanoparticles. Note that the filter may also influence the particle signal when the particle size becomes comparable or larger than the wavelength of light, which complicates relating the optical signal to particle properties in the case of larger particles. For small enough particles such that the filter only affects the background signal, the recorded intensity is then to the lowest order in the scattered field22
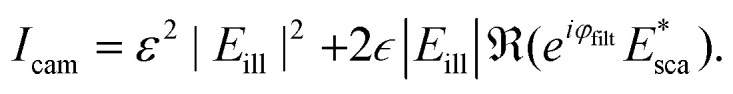


Notice that the first term is quadratic in the attenuation *ε*, while the second term is linear in *ε*. Thus, the relative importance of the two terms can be adjusted by adjusting the transmittance of the filter. However, note that depending on the value of *ε* the contribution of |*E*_sca_|^2^ may no longer be negligible. Thus, the value of *ε* affects which approximation that can be used when relating the scattering signal to particle properties.

A different approach to tune the signal-to-background ratio is to use a reflection rather than transmission geometry, as depicted in Fig. 4C.^65^ This measurement geometry is in the literature typically denoted iSCAT (interferometric scattering).^66^ When using a reflection geometry with transparent coverslips, a small portion of the illuminating light will be reflected back to the camera at the interface between the coverslip and the sample. This optical field constitutes the term *E*_ill_ in this geometry. Most of the light will be transmitted through the interface. This transmitted light produces the scattered field *E*_sca_ from particles in the sample. Denoting the reflectivity of the interface *ε*, the recorded intensity is again identical to eqn (23) if one adds the additional relative phase term *φ*(*z*),23
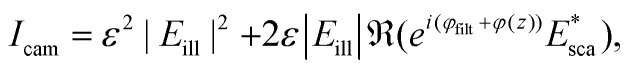
*φ*(*z*) is the relative phase difference between the particle signal and the background signal due to different optical path lengths caused by the measurement geometry. For example, in a reflecting measurement geometry, the recorded particle signal and the background signal may originate from different depth positions within the sample, causing *φ*(*z*) to be non-zero and dependent on the depth position of the particle. In some works, these two approaches for background attenuation have been employed in parallel to achieve maximal sensitivity.^27,60^ Moreover, by using two reflections from the top and the bottom of microfluidic channels, the interference between the two reflections can be used to control the phase and amplitude of the background signal.^23^ Thus, there are several ways of controlling the amplitude of the background signal, which in turn affects the relation between signal and polarizability.

### 2.3 Holographic microscopies

Although interferometric scattering techniques have emerged as powerful techniques for particle characterization with low detection limits, only the real part of the scattered light is detected and quantified. Holographic microscopy, by contrast, achieves quantification of the full complex-valued optical field, thereby providing a more complete characterization of the scattered light, which can be used to distinguish between particles of different materials such as particles-bubbles^14^ and gold-dielectric particles.^67^ Even though holographic microscopy historically has mostly been used to investigate samples such as live cells,^26^ it has recently been shown that it is possible to detect particles down to single proteins when on a surface^28^ and single viruses in solution.^21^

**Fig. 5:**
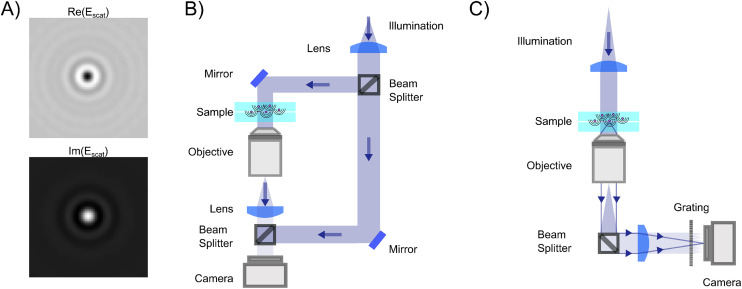
Holographic microscopy setups: (A) in holographic microscopy, both the real (upper) and imaginary parts (lower) of the scattered field are quantified, where the sign and amplitude of the real and imaginary part contain particle material information.^14,67^ (B) Off-axis holography: the illumination light is split into two separate paths prior to the sample. The reference arm and the sample arm are recombined close to the camera at an angle. (C) Quadriwave lateral shearing interferometry (QLSI): the light is split using a diffraction grating after the sample. The grating splits the incoming light into multiple identical light beams that all are slightly shifted and tilted with respect to each other, where all beams interfere with each other at the camera plane.^45^

In holographic microscopy, one or more images of the sample is combined with optical imaging theory to achieve quantification of the real and imaginary parts of the complex-valued optical field itself (Fig. 5A). This can be achieved using several different microscopy configurations, both using a single image and multiple images to obtain the optical field.^45,68^ One commonly employed trick to achieve single-image holographic microscopy that is used in off-axis holography is to introduce an external reference field that incident on the camera at an angle *θ*_ref_ such that sin *θ*_ref_ > 3 sin *θ*_max_/*M* (Fig. 5B), where *M* is the magnification of the optical system. From the interference with the reference field, it is then possible to quantify the optical field and its angular components (Box 5)^69^ Another way to achieve single-image holography is to introduce a grating at the camera plane, such as in QLSI (Fig. 5C), where the complex-valued optical field can be obtained from the resulting interference pattern.^45^ The different implementations have some different strengths and weaknesses regarding complexity, noise, and stability. A detailed discussion about different microscopy configurations can be found in ref. 45.

**Box 5 Quantifying the optical field**

A common way to achieve holographic microscopy is to introduce a reference field at the camera plane, propagating at an angle *θ*_ref_ with respect to the optical axis. Taking the reference field to propagate parallel to the *xz*-plane, the field at the camera plane can be represented as a plane wave as *E*_ref_(*x*,*y*) = |*E*_ref_|*e*^i**k**_ref_·**x**^, where **k**_ref_ = *k* sin *θ*_ref_*x̂*. As a further simplifying assumption, we consider an illumination propagating along the optical axis. In this case, the intensity recorded by the camera reads24
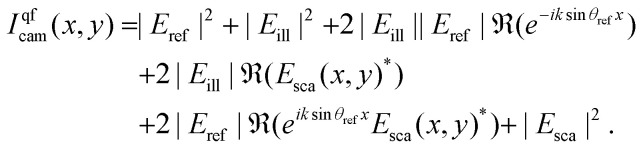

Now, let us investigate the plane wave decomposition (the Fourier transform) of this recorded intensity. Since typically *E*_sca_ ≪ *E*_ill_ and *E*_sca_ ≪ *E*_ref_, we keep only terms up to linear order in *E*_sca_. Further, we utilize that at the camera plane, 25
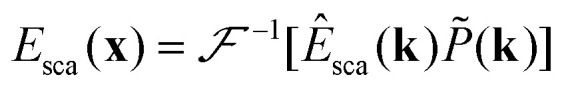
26
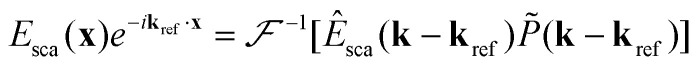
to arrive at27
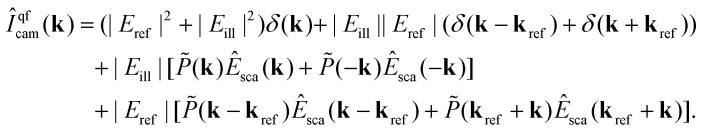

At this point, recall that the function *P̃* has support only for arguments |**k**| < *k* sin *θ*_max_/*M*. It is therefore possible to choose **k**_ref_ such that *P̃*(**k**)*P̃*(**k** − **k**_ref_) = 0 for all values of **k**. Specifically, this holds if sin *θ*_ref_ > 2 sin *θ*_max_/*M*. In this case, one finds that 28
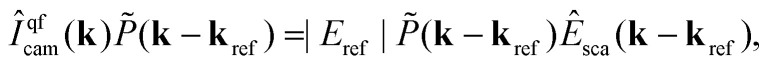
from which the optical field can be reconstructed through an inverse Fourier transform. In this analysis, the term |*E*_sca_|^2^ was neglected. To ensure that *P̃*(**k** − **k**_ref_) does not overlap with this term either, one can show that it is sufficient that sin *θ*_ref_ > 3 sin *θ*_max_/*M*.

Similar to interferometric scattering techniques, optical field measurements can also be combined with optical filters^20,23,64^ and different illumination strategies^28,70^ to improve the detection limit. In that case, the attenuation factor *ε* will affect the relation between the measured particle signal and particle properties.

## 3 Scattering of light from nano- and microparticles

In the preceding section, the optical field was taken to be a scalar quantity to simplify the equations. In reality, the optical field is a vector with components given by the polarization state of the light. The different polarization directions scatter light slightly differently, therefore complicating the description of the scattered light. Nonetheless, as we will show below, under certain circumstances, the scalar description of light still provides an accurate description of light scattering.

The scattering of light from nanoparticles can generally be described by solving Maxwell's equations for electromagnetism with the appropriate boundary conditions. In the special case of spherical particles, the exact solution to this problem was derived by Gustav Mie in 1908.^24^ The field scattered by a homogeneous sphere is written as an infinite sum of special functions, which is numerically much faster to evaluate than explicitly solving Maxwell's equations.

To gain insight into the behavior of the scattered light and into how the light scattering is affected by particle properties, it is useful to consider limiting cases for which the Mie solution can be evaluated analytically. One such limit, which is of particular relevance for nanoparticles, is the *Rayleigh limit*, valid for *kR* ≪ 1, where *k* = 2π/*λ* and *R* is a characteristic size of the particle. Let the illuminating light be a linearly polarized plane wave with amplitude |*E*_ill_|, propagating along the *z*-axis and defining the *x*-axis to lie along the polarization direction. Now, the scattered wave is described both by the angle *θ* relative to the propagation axis of the light and by the angle *ϕ* relative to the polarization axis. The scattered field will have polarization components given by29
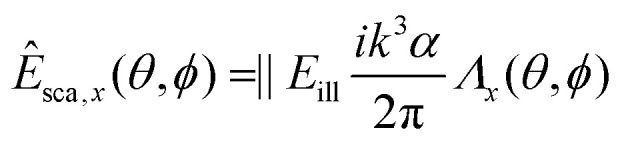
30
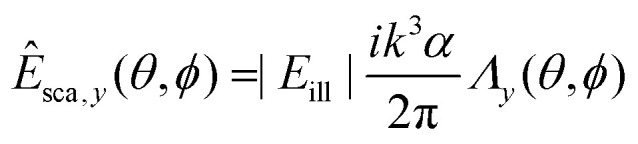
31
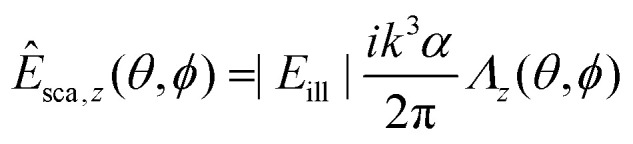
where *α* is the *polarizability* of the particle,32
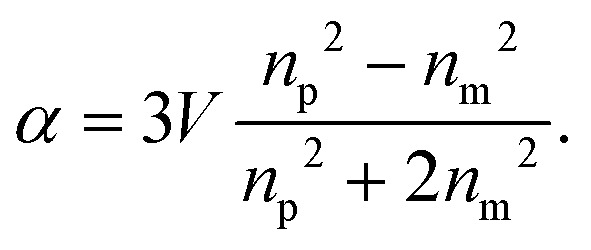


In this expression, *V* is the particle volume, *n*_p_ is the refractive index of the particle, and *n*_m_ is the refractive index of the surrounding medium. The functions *Λ*_*i*_(*θ*,*ϕ*) encode how the polarization of the scattered light is related to the polarization of the illuminating light. They are given by

33 *Λ*_*x*_(*θ*,*ϕ*) = cos^2^ *θ* cos^2^ *ϕ* + sin^2^ *ϕ*

34 *Λ*_*y*_(*θ*,*ϕ*) = (1 − cos^2^ *θ*) cos *ϕ* sin *ϕ*

35 *Λ*_*z*_(*θ*,*ϕ*) = cos *θ*sin *θ*cos *ϕ*.

The polarization states propagate to the camera independently, so that their contribution to the signal at the camera can be obtained through superposition. For readability, we will in the following only consider the contribution of *Λ*_*x*_ to the signal at the camera. Furthermore, focusing on spherically symmetric nanoparticles we can integrate out the dependence on the azimuthal angle *ϕ* to write *Λ*_*x*_ = π(cos^2^ *θ* + 1). The other polarization directions can be treated analogously and are explicitly considered in the ESI.†

Another useful limit is the *weakly scattering limit*, that is, for *kR*|*n*_p_/*n*_m_ − 1| ≪ 1 and |*n*_p_/*n*_m_ − 1| ≪ 1.^24^ This is a slightly weaker condition than the Rayleigh condition, and the corresponding approximation is valid also for arbitrarily large scatterers as long as the refractive index difference compared to the surrounding medium is sufficiently small so that the inequality *kR*|*n*_p_/*n*_m_ − 1| ≪ 1 still holds. This limit is particularly useful for biological nanoparticles, which typically obey these inequalities. In this case, the polarizability is typically approximated as

36 *α* ≈ 2*V*Δ*n*/*n*_m_,

where Δ*n* = *n*_p_ − *n*_m_. In the specific case of biological nanoparticles, this expression has a particular physical interpretation since it enables the treatment of the nanoparticle as a volume made up of biomolecules at a certain concentration. The refractive index of a solution of biomolecules increases approximately linearly with the mass concentration *C* of molecules, as *n*_p_ = *n*_m_ + (d*n*/d*c*)*C*, where (d*n*/d*c*) is called the specific refractive index increment, and is material specific. However, since most biomolecules contain similar elements at similar ratios (mostly carbon, hydrogen, oxygen, and nitrogen), the specific refractive index increments of different types of biomolecules are very similar.^26^ Typical values range from ∼0.16 ml g^−1^ for carbohydrates to ∼0.2 ml g^−1^ for nucleic acids and proteins.^26^ The polarizability times *n*_m_ thus evaluates to *n*_m_·*α* ≈ 2(d*n*/d*c*)*C*·*V* = 2(d*n*/d*c*)*m*, where *m* is the total mass of the biomolecules in the nanoparticle. Thus, in the case of weakly scattering particles, the polarizability is proportional to particle mass.

Further, the total scattered field can be calculated as the superposition of the field scattered by infinitesimal volume elements within the particle. The scattering from each such infinitesimal volume element is given by the Rayleigh scattering limit above. For weakly scattering particles, the optical field impinging on each such volume element can be approximated as equal to the incident optical field external to the particle. The scattered field from an isotropic particle, evaluated outside of the particle, is then given by ref. 7137
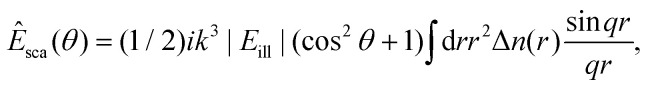
where *q* = 2*k* sin(*θ*/2). This approximation is called the Rayleigh-Debye-Gans (RDG) approximation.

The integral above describes the interference of the light scattered from different volume elements in the particle. This factor is denoted the *form factor* and physically encodes the distribution of refractive index within the particle. The RDG field is often written in the following form,

38 *Ê*_sca_(*θ*) = (1/2)*ik*^3^|*E*_ill_|*αΛ*_*i*_(*θ*)*f*(*θ*;*R*),

where *f*(*θ*;*R*) is the form factor, which for an isotropic particle depends on its size *R* and internal refractive index distribution.

Under uniform illumination, analytical solutions to the form factor within the RDG approximation can be attained for some specific geometries, such as spheres and core–shell spheres. For a spherical particle, the form factor is,^7^39
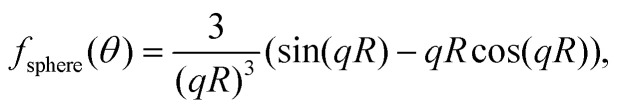
and for an infinitesimal spherical shell, which can be used to approximate the signal from a lipid vesicle, the form factor is^72^40
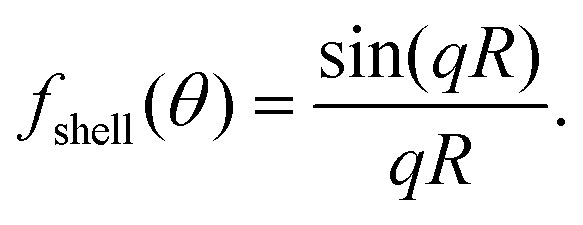


**Fig. 6:**
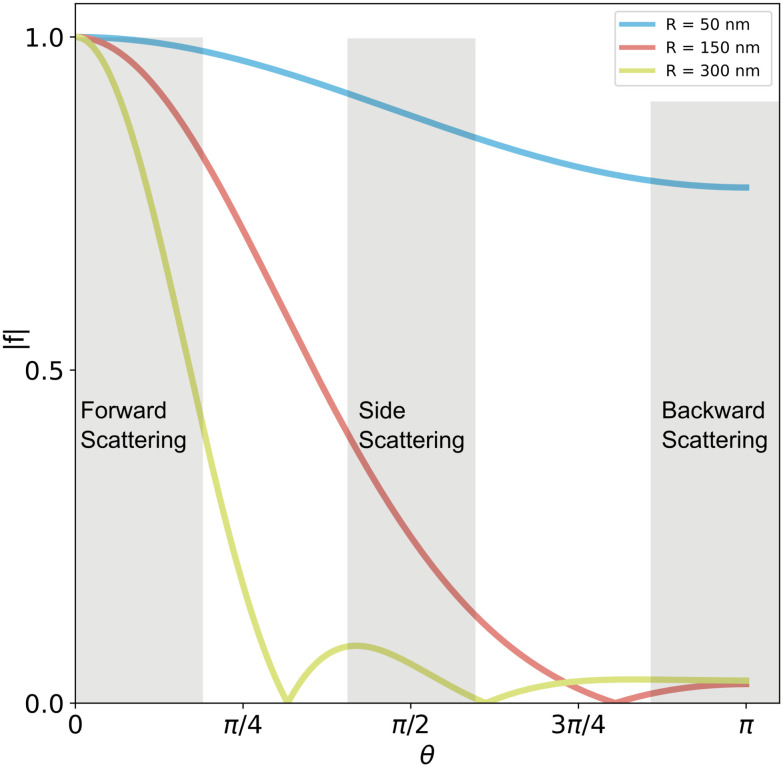
Form factors of homogeneous spheres. The form factor depends strongly on the particle size. Small particles (blue line) scatter almost uniformly, while large particles (green line) scatter predominantly in the forward direction (small angles). In forward scattering, the contribution from the form factor is close to unity in most cases. In side scattering (*θ*_ill_ ≈ π/2), the contribution is more pronounced, and in backward scattering (*θ*_ill_ ≈ π), the form factor contribution is maximal. The form factors in this plot are calculated for homogeneous spheres in water illuminated with a wavelength of *λ* = 532 nm. Reducing the illumination wavelength will compress all scattering curves to smaller scattering angles. Conversely, for longer illumination wavelengths the scattering curves will be extended to the right in the figure above.

Note that the form factor at 0 degree scattering angle is identically equal to unity (*f*(0) = 1) and that the form factor for all scattering angles *θ* > 0 is smaller than unity (*f*(*θ* > 0) < 1). For this reason, the relation between the measured optical signal and particle size is different for different measurement geometries. This is highlighted in Fig. 6, in which the form factor of spheres of different sizes is shown as a function of the scattering angle. In accordance with the discussion above, the form factor is close to one for all particle sizes for transmission geometries, for which the scattering angle is close to *θ* = 0. For side-scattering (exemplified in Fig. 3C) and backward scattering (exemplified in Fig. 4B), the form factor greatly influences the relationship between the measured optical signal and particle size. Specifically, the contribution from the form factor to the scattered light becomes appreciable for particles with *qR* > 1. Recalling that *q* = 2*k* sin(*θ*/2), one has that the form factor contribution is appreciable if *R* > (2*k* sin(*θ*_ill_/2))^−1^. For an illumination wavelength of 532 nm when the particle is in water (*n*_m_ ≈ 1.33), this amounts to *R* > 45 nm for side-scattering with *θ*_ill_ = π/2, and *R* > 30 nm for backscattering with *θ*_ill_ = π.

Moreover, although this tutorial focuses on isotropic particles or particles such as aggregates that can be well approximated as isotropic particles,^71^ not all particles are isotropic. For anisotropic particles such as ellipsoids and anisotropic structures such as microtubules, the polarizability is a tensor, causing both the polarization and amplitude of the scattered light to depend on the orientation of the particle.^73–75^ Thus, when relating the signal from anisotropic particles to particle properties, it is important to consider experimental details such as the exposure time and polarization of the illumination signal. Regarding exposure time, if the exposure time is shorter than that of rotational diffusion, the optical signal will fluctuate around an average value.^73^ Regarding polarization, interference can only occur between light of the same polarization. Thus, for methods such as interferometric scattering and holographic microscopies, where the measured signal often is the interference between the particle signal and a background signal, it is primarily the scattered light of the same polarization as the incoming light that will be measured. This dependence on particle orientation and polarization can be suppressed when illuminating the sample with circularly polarized light, which is why interferometric scattering methods such as iSCAT most often illuminate the sample with circularly polarized light.^75^ Thus, the optical signatures from anisotropic particles can be magnified and reduced based on the optical setup and experimental design.

## 4 Relation between the signal measured in a elastic scattering microscope and physical particle parameters

Now, let us use the mathematical framework developed in sections 2 and 3 to investigate the relation between the scattered light from a nanoparticle and the optical field at the camera plane. The crucial insight is that the angular distribution of the scattered field from a nanoparticle described in section 3 describes precisely the individual plane wave components used in eqn (4). Working with isotropic particles within the RDG approximation while also assuming that the illuminating light propagates along the optical axis, we write the scattered field as

41 *E*_sca_(*θ*,*ϕ*) = (1/2)*i*|*E*_ill_|*k*^3^*αf*(*θ*)(cos^2^ *θ* + 1),

where *α* is the polarizability of the particle, *f*(*θ*) is the form factor, |*E*_ill_| is the amplitude of the illuminating light. For a particle located in the focal plane of the objective (see Box 6 for a description of the general case of a particle located away from the focal plane), the scattered field at the back focal plane is then given by42
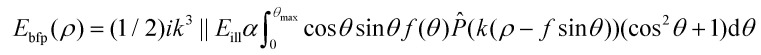
and the scattered field at the camera plane is given by43
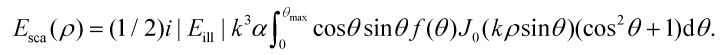

**Box 6 Effect of having particle located away from the focal plane**

Eqn (43) is valid for a scatterer located at the focal plane of the objective. In an experiment, this condition is not necessarily fulfilled. If the particle is located a distance *z* from the focal plane, the individual plane wave components of the scattered field must additionally be propagated to the focal plane. Following the logic in Box 3, since each of the components describes a plane wave propagating at an angle *θ* with respect to the optical axis, the angular components propagated a distance *z* along the optical axis are given by

44 *Ê*_cam_(*θ*,z) = *Ê*_cam_(*θ*,0)*e*^*ikz*cos *θ*^.

Thus, the scattered field from a nanoparticle located a distance *z* from the focal plane will, at the camera plane, reads 45
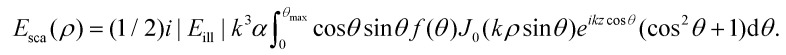

We will now use this expression, in combination with the definition of the RDG form factor eqn (37), to investigate how the signal measured in a elastic scattering microscope is related to the physical parameters of the scattering objects.

### 4.1 Darkfield microscopies

In the case of darkfield microscopy, the intensity measured by the camera from a particle located at the front focal plane of the objective and illuminated by a plane wave propagating along the optical axis is given by46
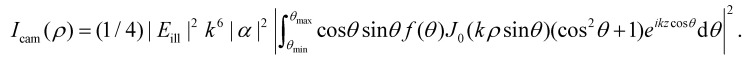


**Fig. 7:**
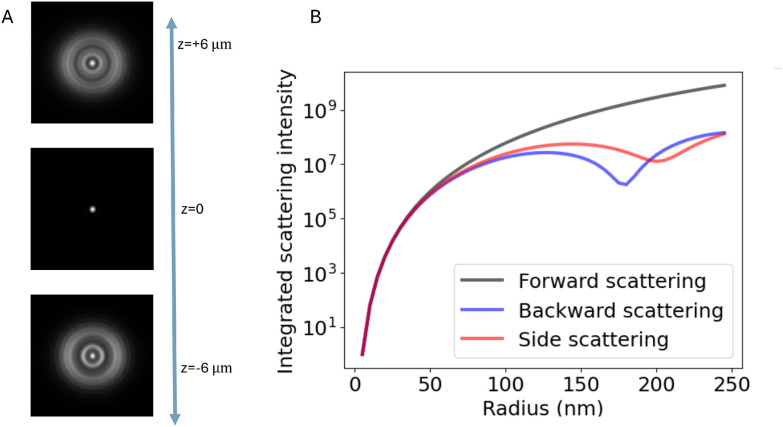
Scattered intensity in darkfield imaging (A) calculated microscopy images of nanoparticles (radius 20 nm) suspended in water and measured in darkfield microscopy with *θ*_ill_ = 0 and wavelength 532 nm at different values of the depth positions *z*. The field of view of each particle image is 10 × 10 microns. (B) The integrated intensity of microscopy images measured in darkfield microscopy as a function of particle size for three illumination angles. For *θ*_ill_ = 0, the integrated intensity scales with the square of the volume throughout the sizes included in this calculation. For *θ*_ill_ ≠ 0, distinct minima appear corresponding to the minima of the form factor.

Some examples of darkfield microscopy images obtained from eqn (46) at different depth positions *z* are shown in Fig. 7A.

To perform particle characterization, one needs to reduce the measured scattering to a set of values representing some physical properties of the particle. The most common way of achieving this is by characterizing the integrated intensity of a scattering pattern in a microscopy image, which in the case of darkfield microscopy is proportional to the square of the polarizability. In the case of a particle illuminated by a plane wave with *θ*_ill_ = 0, one obtains for an isotropic scatterer (see Box 7 for a derivation of this result)47
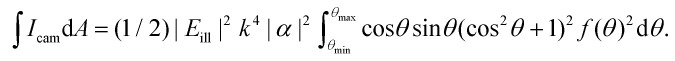

**Box 7 Integrated darkfield intensity**

In order to calculate the integrated scattering intensity in darkfield microscopy, it is useful to start with the expression

48 *I*_cam_(**x**) = |*E*_sca_(**x**)|^2^.

Integrating this over the entire detector surface, one has 49
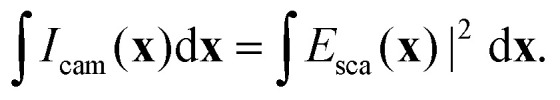

Now, we invoke Parseval's theorem, stating that 50
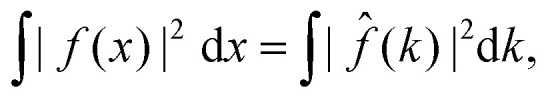
where *f*(*x*) and *f̂*(*k*) are Fourier transform pairs. One therefore has51
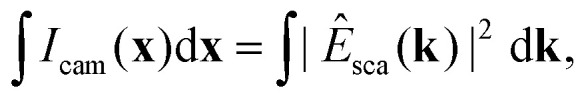
from which eqn (47) follows after transformation into polar coordinates and switching integration variable from the wave vector |**k**| to angle *θ*.

Similar expressions for cases in which the illuminating field is not parallel to the optical axis can be derived by adjusting the limits of integration in the above expression. Furthermore, for small enough scatterers such that *f*(*θ*) ≈ 1, the integrated darkfield intensity is proportional to the square of the polarizability. For larger particles (*kR* > 1), the contribution to the measured signal from the form factor of the scatterer will depend on their size and morphology.^76^ Note that here only the component of the polarization parallel to the polarization of the illuminating light is considered. In a configuration where all light on the camera is collected, the perpendicular polarization directions contribute another factor of 2 to this result, as explicitly derived in the ESI.†

The approaches to achieve darkfield illumination discussed in section 2 give rise to slightly different contributions from the form factor. For darkfield imaging through spatial blocking, the illumination angle *θ*_ill_ = 0 in eqn (47). In the case of darkfield illumination by oblique illumination, one instead has*θ*_ill_ ≠ 0. The final case of evanescent wave scattering is slightly more complicated. In this case, the optical field is propagating along the interface in which total internal reflection occurs. Therefore, the illumination angle is *θ*_ill_ = π/2. However, the form factor is slightly different since the optical field decays exponentially in the medium of the scatterers. The appropriate correction was derived in the ESI of ref. 76. In Fig. 7B, the integrated scattered intensity is shown as a function of particle size for a fixed refractive index for the specific cases *θ*_ill_ = 0, *θ*_ill_ = π/2 and *θ*_ill_ = π. As discussed in section 3, the larger the *θ*_ill_, the smaller the size region for which there is a unique relation between scattering signal and size for a known particle refractive index.

### 4.2 Interferometric scattering microscopies

In the case of interferometric scattering microscopy techniques, the term of interest in eqn (15) is the final interferometric term. Taking the illuminating field to be a plane wave propagating along the optical axis, the integrated recorded intensity is given by (see Box 8 for a derivation)52
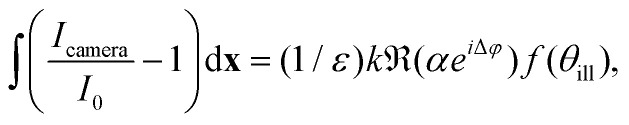
where Δ*φ* = *φ*_filt_ + *φ*(*z*) is the relative phase difference between the background field and the scatterer, *ε* is the attenuation factor, and *φ*(*z*) describes the depth-dependent relative phase shift of the particle relevant for reflection geometries.^77^ Further, we have defined *I*_0_ = *ε*^2^|*E*_ill_|^2^, which is experimentally estimated by evaluating the intensity recorded by the camera in locations without particles present. Note that the right-hand side of this final expression does not depend on the intensity of the illuminating light. In other words, the illuminating light does not need to be separately quantified to perform particle characterization in interferometric scattering approaches. However, since *ε* may have a spatial dependence or vary between different surfaces,^23,75^ background corrections are still sometimes needed. Also, in contrast to darkfield approaches, the recorded intensity is now directly proportional to the real part of the polarizability when Δ*φ* = 0.

**Box 8 Integrated interferometric intensity**

To evaluate the integrated signal from a particle in interferometric microscopy, we write the illumination field at the camera plane as

53 *Ẽ*_ill_ = *ε*|*E*_ill_|*e*^*i*(*φ*_filt_±*kz*)^,

where *ε* is the attenuation coefficient of the illuminating light, *φ*_filt_ is the phase shift of the illuminating field compared to the scattered field caused by the presence of optical filters, and *z* is the distance from the scatterer to the focal plane. The ± reflects the fact that the light propagates in opposite directions compared to the original propagation direction for reflection and transmission geometries. The + sign is relevant for reflection geometries, and the − sign for transmission geometries. To the lowest order in the scattered field, the recorded intensity, in the RDG approximation and assuming an isotropic scatterer, is54
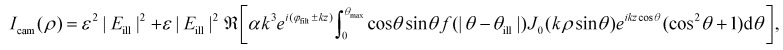
where *θ*_ill_ = 0 for interferometric imaging with transmission geometry, and *θ*_ill_ = π for interferometric imaging with reflection geometry. The first term in this equation represents the intensity recorded by the camera in the absence of scatterers. Subtracting and dividing by this value, one obtains for the integrated signal of a particle55
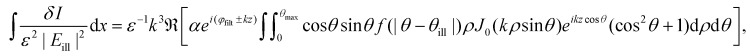
where *δI* = *I*_cam_ − *ε*^2^|*E*_ill_|^2^. To evaluate this, recall that the integral over *ρ* is a Fourier transform in disguise. Utilizing that56
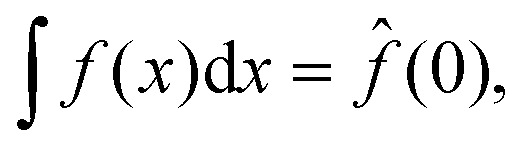
where *f* and *f̂* are Fourier transform pairs, one immediately finds57
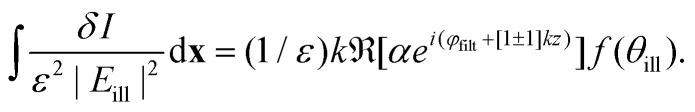

**Fig. 8:**
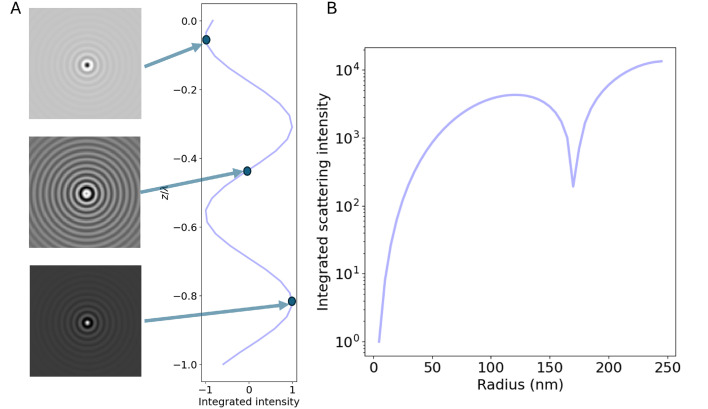
Scattered intensity in interferometric imaging (A) calculated microscopy images of nanoparticles (radius 20 nm) suspended in water and measured in interferometric microscopy in reflection geometry (*θ*_ill_ = π) with illumination wavelength 532 nm at different values of the depth position *z*, indicated in the plot to the right of the microscopy images. The integrated intensity of the microscopy images varies sinusoidally with the depth position *z* due to the phase shift *φ*. The field of view of each particle image is 10 × 10 microns. (B) The integrated intensity of microscopy images measured in interferometric microscopy with *θ*_ill_ = π as a function of particle size.

As noted previously, only the polarization component parallel to the polarization of the illuminating light is considered in this expression. However, only this component will interfere with the illuminating light and contribute to the interferometric signal. Thus, no additional contributions to the interferometric signal need to be considered. To exemplify the position dependence of the interferometric scattering signal, in Fig. 8A microscopy images calculated according to eqn (54) in the case for interferometric backscattering. Notice how the central lobe changes sign as the depth position changes by only a fraction of a wavelength. The integrated signal of the microscopy images shows a sinusoidal dependence on the depth position *z* due to the relative phase difference *φ*(*z*) between the scattered light from the particle and the light reflected at the coverslip. For this reason, the iSCAT signal is either quantified in the same plane for all particles, as on a coverslip or a specific depth plane,^15,27^ or the images are transformed using a neural network to remove the depth dependence.^23^ When that is done accurately, the size dependence of the integrated signal follows Fig. 8B, which shows eqn (52) when Δ*φ* = 0.

### 4.3 Holographic microscopies

**Fig. 9:**
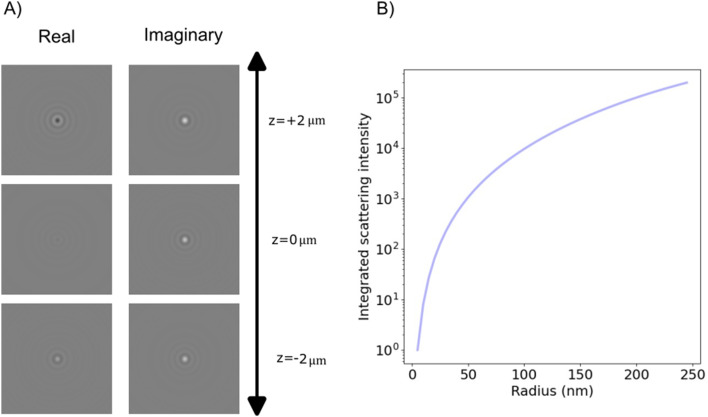
The obtained complex-values particle images in holographic imaging (A) calculated microscopy images (real and imaginary parts) of nanoparticles (radius 20 nm) with a real-valued refractive index suspended in water and measured in holographic imaging with illumination wavelength 532 nm at different values of the depth position *z*. Note that the images share the sample color scale where the white and black colors, respectively, correspond to positive and negative optical field values compared to the background illumination. When in focus (*z* = 0), the integrated particle signal in the imaginary-part image is related to the real part of the particle polarizability (eqn (58)), which also is related to the phase shift caused by the particle. The integrated particle signal in the real-part image is in turn related to the imaginary part of the particle polarizability (eqn (58)), which related to light absorption. Thus, for a particle with a real-valued refractive index, the real part of the optical field is approximate zero for particles when in focus (eqn (58)) and the sign of the real part of the optical field changes at different sides of the focus as a result of the Gouy phase. The field of view of each particle image is 10 × 10 microns. (B) When the particle is in focus, the integrated imaginary part of microscopy images is measured in holographic microscopy scales with particle volume. Note that for holographic imaging, the image can be re-propagated after recording. This makes it possible to refocus the detections individually.

In holographic microscopies, the recorded quantity is the scattered field itself. In this case, one has that^67^58
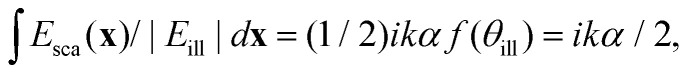
where the last equality is valid for *θ*_ill_ = 0, which is the most common measurement geometry for optical holographic methods. This is similar to eqn (52) with *φ* = 0, except for the fact that the polarizability is now allowed to be complex-valued. Note that the integrated imaginary part of the optical field is proportional to the real part of the particle polarizability, which can be used to distinguish between particles of different materials.^14,67^ Moreover, in the case of biological particles, when eqn (36) is inserted into eqn (58), the same formula as for the integrated signal from cells using quantitative phase microscopy is obtained,^78^ which highlights the correspondence between holographic imaging of nanoparticles and quantitative phase microscopy of cells. In Fig. 9A, the real and imaginary parts of the microscopy images measured in holographic imaging are shown for different depth positions, where in focus, the integrated particle signal in the imaginary-part image is related to the real part of the particle polarizability and the integrated particle signal in the real-part image is related to the imaginary part of the particle polarizability (eqn (58)). Importantly, following Box 3 and eqn (44) the optical field signal can be re-propagated after recording the image. This, in turn, enables quantification of the signal of focused scattering patterns even though they are measured out of focus, which reduces the sensitivity of the particle characterization to noise and out-of-focus effects.^67^ The integrated imaginary part of the signal is proportional to particle polarizability and hence particle volume (Fig. 9B) as predicted from eqn (58).

Furthermore, utilizing eqn (12) one can rewrite the scattered field at the camera plane in terms of the angular components of the form factor directly (Box 9). This shows how holographic imaging contains information about particle polarizability and the particle form factor itself, which, if quantified, can be related to particle size.^8^

**Box 9 Quantifying the optical form factor in holographic imaging**

The scattered field at the camera plane is related to the optical form factor as59
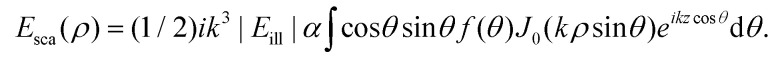

Applying the transform eqn (12), one finds that 60
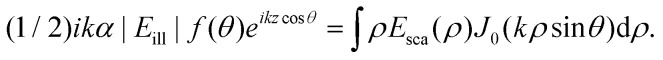

Taking the absolute value of both sides and utilizing that |*e*^*ix*^| = 1, one finds 61
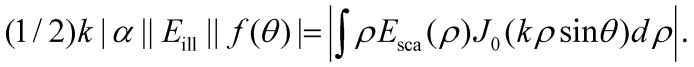

Finally, since 
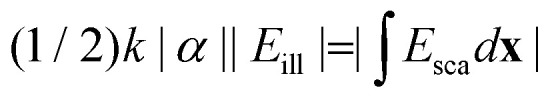
 from eqn (58), we have that 62
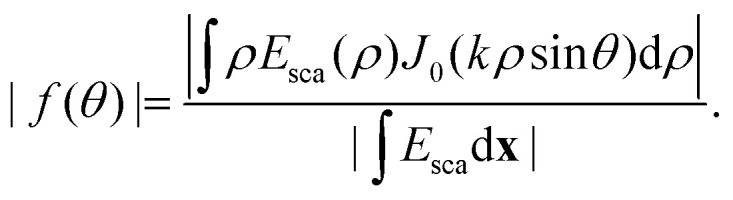

## 5 A toolbox for analyzing elastic scattering microscopy data

Quantitative analysis of elastic scattering microscopy data consists of two fundamental steps, namely *particle detection* and *signal characterization*.^79^ This section aims to provide an easy-to-use toolbox to perform these tasks for the three types of elastic scattering microscopy geometries that are discussed in this tutorial. If the particles are moving during the experiment, a third step, *detection linking*, is required to form particle traces,^80^ allowing the particle motion to be related to particle properties such as the hydrodynamic radius as previously described in several review articles.^17,81^ Since the ability to track the motion of particles is generic for optical microscopy methods, the focus of this tutorial is the information contained in the optical signal and how to extract this information using deep learning, where the hydrodynamic radius will complement the optical signal in the case of freely suspended particles. Appended to this tutorial are Jupyter notebooks containing code for performing particle detection and characterization in the three scattering modalities considered here (darkfield imaging, interferometric imaging, and holographic imaging). Unlike other toolboxes that only simulate optical particle scattering,^82,83^ these notebooks go beyond by combining simulations with deep learning image analysis. In the following two sections, the content of the notebooks will be briefly explained.

### 5.1 Particle detection

The particle detection task is essentially recognizing scattering patterns in microscopy images in the presence of noise. Traditionally, particle detection has been performed by algorithmic approaches, in which a predefined set of image filters are applied to the microscopy images, followed by a thresholding operation to identify particles.^84^ In the past decade, deep learning approaches to particle detection have become increasingly popular, showcasing more accurate detection in particular under low signal-to-noise conditions.^79,85^

There are, in general, three sources of noise contributing to the noise level in elastic scattering microscopy images: (1) *shot noise*, arising from the finite number of photons detected in each camera pixel, (2) *read noise*, which is intrinsic to the camera, and (3) *speckle noise*, due to coherent reflections and scattering along the beam path of the illuminating light.^86^

The first two noise sources are common to all types of microscopy and have the property of being spatiotemporally independent: the noise at pixel *i* at time *t*_0_ is independent of the noise at pixel *j* at time *t*_1_. This particular property means that the noise from these sources can be reduced by averaging the signal over time and/or across multiple pixels. Speckle noise is special for optical microscopy and originates from the interference between the different optical plane waves of the illumination. Speckle noise is characterized by the fact that noise at neighboring pixels is correlated, where the amplitude and temporal stability of the speckle depends on the light source and experimental setup.^87^ Important for image analysis, this noise has a spatial correlation that is similar to the spatial correlation of the nanoparticle scattering signal, and in the absence of mechanical vibrations in the system, it can be considered static. Thus, neither temporal nor spatial averaging helps to reduce the effect of this particular noise term. This noise source is primarily important for interferometric approaches, where the particle contrast is determined relative to the illuminating optical field.^87^

To improve the detection limit, both background subtraction and signal averaging are commonly used, where the approach depends on whether the particles are immobilized or freely diffusing. For freely diffusing particles, the background can be subtracted by averaging adjacent frames to the current frame as the background features are static and the particle of interest is moving.^14,15,23^ When working with such background subtraction, it is important that the background frames are chosen so that the particle signal is not subtracted. Moreover, for freely diffusing particles, it is difficult to improve the detection by averaging frames, as the particles are at different positions in each frame. A special case is when looking at particles binding to a surface, where both a rolling background subtraction and frame averaging can be applied.^60^ For immobile particles, frame averaging can be used to improve the detection limit,^60,88^ where the background subtraction is typically done by using images prior to the particle binding.^58^

**Fig. 10:**
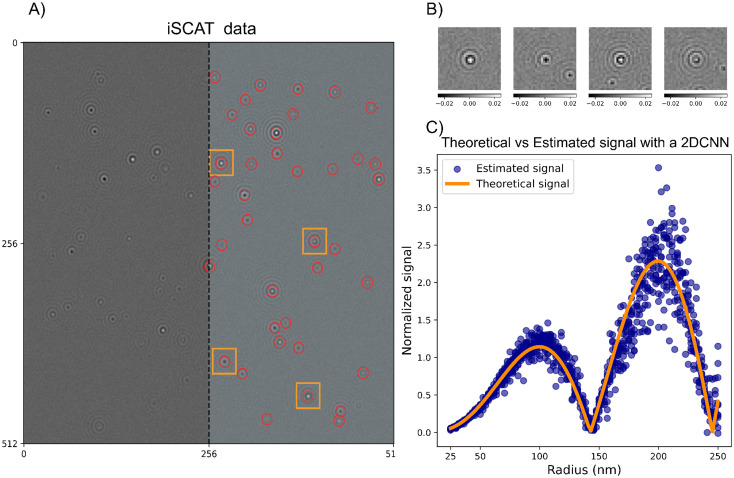
Particle detection and characterization in interferometric microscopy (A) an example iSCAT microscopy image containing multiple scatterers. This image was simulated using the notebooks appended to this tutorial. On the right side of the image, particle detections made by the LodeSTAR algorithm, trained using the notebooks appended to this tutorial, are overlaid on the image. The particle detections are intentionally left out on the left half of the image to not obscure the appearance of the scattering data. (B) Crops of the microscopy images of individual particles detected in the frame in (A). (C) Characterization of the integrated scattering intensity of individual particle crops like the ones shown in (B). The characterization is performed by a convolutional neural network, trained using the notebooks appended to the tutorial.

After background subtraction, the particles are often readily detectable using several different methods. In the notebooks, particle detection using both deep learning analysis (specifically LodeSTAR^79^) and an algorithmic approach (radial variance transform^15^) is demonstrated on simulated images of scattering patterns for the different modalities. An example of the particle detection step using LodeSTAR is shown in Fig. 10A for the case of interferometric imaging with reflection geometry. To handle the change of signal for different particle depth positions, the network is trained on a range of different *z*-positions, which enables accurate detection of the particles in the image.

### 5.2 Signal characterization

After having detected a particle, the next step in nanoparticle characterization is to utilize the image of the scattering pattern to extract information about the particle itself.

Common to all elastic scattering microscopy approaches is that the integral of the particle signal is related to particle polarizability (which, for biological nanoparticles, is proportional to their mass). However, depending on the measurement geometry, when relating the scattering to mass, the effect of the optical form factor needs to be compensated (Fig. 6). As a rule of thumb, for particle sizes *R* < (2*k* sin *θ*_ill_)^−1^, the form factor can be approximated as *f*(*θ*) ≈ 1 within the angles collected by the objective, in which case the integrated intensity is directly related to particle polarizability. For particles larger than this, the integrated intensity must be complemented with independent measurements of size and/or polarizability to perform quantitative characterization, as exemplified in.^23,89^

In practice, the task in signal characterization is to estimate the integrated particle signal in the presence of noise. Directly summing up all camera pixels is not a good approach in practice since, most commonly, tens or hundreds of particles are present in the field of view of the camera at the same time. The most common approach to signal characterization is, therefore, to crop out a small region around each detected particle (Fig. 10B), and fit some kind of function to this limited view of a particle. As a specific example in the case of iSCAT, in^89^ the particles were first localized using the radial variance transform, where the particle detection with the maximum positive contrast estimated by Gaussian fitting was used to estimate the particle signal.

Another approach that has gained increasing attention is to utilize deep learning enhanced analysis techniques to not only detect the particle but also estimate the signal. In the appended notebooks, we provide code to train and apply a convolutional neural network to estimate the integrated signal strength of scattering patterns in microscopy images. In Fig. 10C, this step is shown for interferometric imaging in reflection geometry, where the signal estimation follows what is expected from theory. In the appended notebooks, code is also provided to perform this step for darkfield imaging as well as for holographic imaging.

Going beyond particle polarizability, it is, in some instances, possible to quantify also the particle form factor directly from the optical signal using deep learning image analysis by utilizing the fact that the form factor is encoded in the angular components of the scattered field as measured in quantitative field imaging (eqn (62)). This approach was utilized in ref. 19 to estimate particle size and refractive index directly from scattering patterns measured in off-axis holographic images. Specifically, particle size (or, more accurately, the radius of gyration of a particle) is related to its scattering form factor as when *Rq* ≪ 1 ^90^

63 *R*_g_^2^ ≈ 3*q*^−2^(1 − *f*(*θ*)^2^).

Thus, the task of particle sizing directly from holographic images amounts to estimating a curvature in the optical form factor from noisy images.

**Box 10 Applying the notebooks to your own data**

Below are general instructions on how to adapt the appended Jupyter notebooks to this tutorial to your own microscopy images.

1. Prepare your data:

 • Collect your data and save it as numpy arrays with a .npy extension.

 • For best results, crop the frames to dimensions that are powers of 2, as the LodeSTAR detection model requires the data to be downsampled twice.

 • The notebook is designed to handle a single frame by default; to process multiple frames, incorporate a for loop to apply detection across each frame.

 • Data normalization (centering around 0) is recommended to improve consistency, though it's not strictly required.

2. Training the LodeSTAR detection model:

 • Begin by selecting at least one cropped region containing a single particle, ideally one that is clearly visible and distinguishable from others. This particle should be representative of those you aim to detect.

 • Suitable sizes for the Region of Interest (ROI) are 32 × 32, 48 × 48, or 64 × 64 pixels.

 • If detection performance is suboptimal, consider including additional particles to increase data complexity, adjusting augmentation settings, or refining the thresholding step for full-frame particle detection. As a tip, do not include too many additional particles; a range of 1–5 should work well.

 • The default settings in the notebooks provide a solid starting point but may require fine-tuning to match your specific data.

3. Training a convolutional neural network (CNN) for detection and signal estimation:

 • For training a CNN to detect and estimate particle signals, start with the neural network architecture provided in the notebook.

 • Modify the data simulation parameters to match your experimental setup. Update variables such as wavelength, resolution, and Numerical Aperture to match your optical system, and set the appropriate range for radius and refractive index based on the particles in your dataset.

 • As a tip, begin with the model provided in the notebooks, then retrain it to fine-tune it specifically for your dataset.

## 6 Considerations when designing measurement geometry

From the treatment above, it is clear that the different approaches to elastic scattering microscopy have different quantitative power when it comes to particle characterization.

The first consideration that one should make when designing a scattering-based characterization experiment is the level of detail required in the characterization to answer the scientific questions at hand. In some cases, it may be sufficient to detect and track the motion of particles rather than accurately quantify the particle signal. In this case, darkfield techniques have the advantage that the data is relatively easily analyzed since the particles appear bright against a dark background. For this reason, darkfield imaging is one of the standard techniques for tracking suspended nanoparticles.^13,51^

**Fig. 11:**
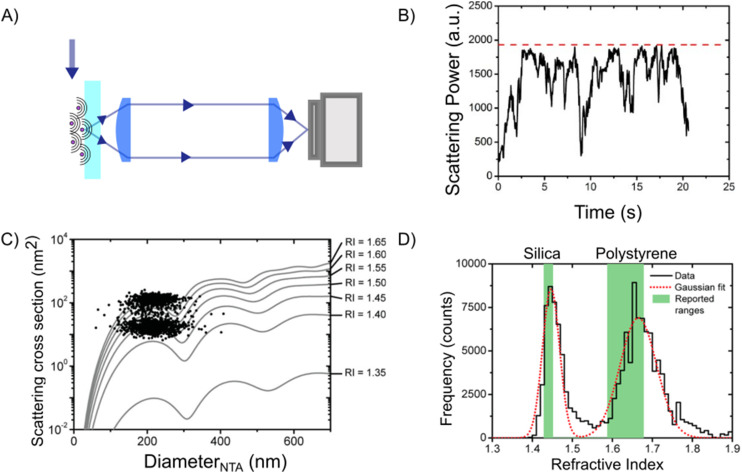
Particle characterization using darkfield imaging with oblique illumination (A) in ref. 13 darkfield imaging with oblique illumination was used for quantitative characterization of suspended nanoparticles. (B) The particle signal was estimated by tracking the motion of nanoparticles and estimating the integrated signal at each time point in a particle trace. The maximum value of the integrated signal was used as a proxy for the signal strength. (C) Using both signal quantification and particle tracking over time enabled quantification of both hydrodynamic size and the scattering cross-section, here for a sample of silica beads and a sample of polystyrene beads. (D) By combining the particle size and scattering cross section the particle refractive index was also estimated for the silica and polystyrene beads. Figure reprinted with permission from American Chemical Society (Copyright 2014).

However, particle characterization based on the optical signal using darkfield techniques is comparatively challenging since relating particle signal to polarizability requires accurate calibration. In particular, since the particle contrast in darkfield techniques is proportional to the local light intensity at the particle position, a proper calibration procedure would require mapping out the illumination intensity throughout the entire field of view, which is technically challenging. In ref. 13, particle characterization was demonstrated using darkfield imaging with oblique illumination (Fig. 11A) of a sample freely diffusing in a macroscopic volume. The challenge of quantifying the scattered signal in such conditions, in particular under a non-uniform illumination, was overcome by utilizing the maximum value of the measured scattering signal of each particle trace as a proxy for the particle scattering, in combination with careful calibration (Fig. 11B). This enabled the quantification of hydrodynamic size as well as the scattering cross-section of suspended polystyrene and silica beads (Fig. 11C), which was also converted into the estimate for the refractive index of these particles (Fig. 11D).

**Fig. 12:**
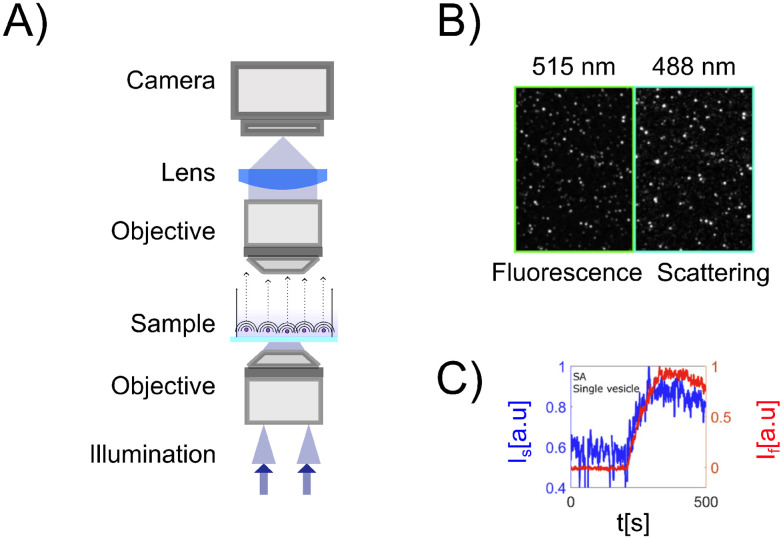
Characterization of surface-bound particles using evanescent illumination (A) in ref. 58 evanescent illumination was used to study protein binding to lipid vesicles adsorbed to a surface. (B) Using a dichromatic mirror, the scattering and fluorescence signals can be simultaneously recorded. (C) The protein adsorption event could be resolved by monitoring the integrated fluorescence and scattering intensities as a function of time on the single particle level. Figure reprinted with permission under the CC-BY license.

Another measurement consideration is whether particle dynamics information is critical or not. To follow the same particle over time it needs to be confined, which can be achieved by, for example, tethering the particle to a surface.^58^ In particular, by using evanescent illumination (Fig. 12A), only particles that are adsorbed or very close to the surface will be illuminated and scatter light. In ref. 58, evanescent illumination was used to study protein adsorption to lipid vesicles adsorbed to a surface. Both fluorescence and scattering signal were measured simultaneously (Fig. 12B), enabling time-resolved monitoring of the adsorbed protein mass to individual vesicles and correlating the scattering signal to fluorescence signal (Fig. 12C).

A third measurement consideration is whether the particle signal must be accurately related to particle properties such as mass. Interferometric scattering approaches have the advantage compared to darkfield techniques in that the particle contrast is measured relative to the local illumination intensity so that the particle estimate is insensitive to changes in the illuminating light intensity. This enables accurate quantification of the scattering signal that can be related to particle properties in a precise manner.^15,16,27^ Nonetheless, measuring the scattering from well-characterized calibration particles is still necessary to calibrate the attenuation factor *ε* and the relative phase difference *φ*. For suspended particles, which diffuse in three dimensions, the particles will quickly explore a volume sufficiently large to cover all possible values of *φ*, rendering calibration of this phase unnecessary in this case.

**Fig. 13:**
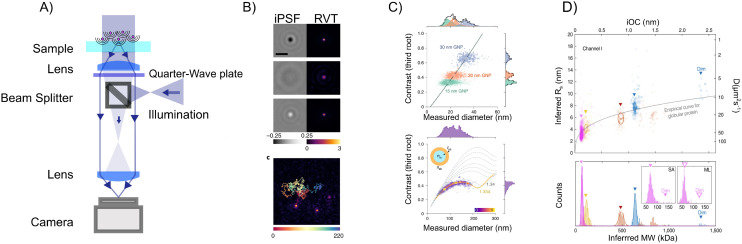
Particle characterization using iSCAT and nanochannel scattering microscopy (NSM). (A)–(C) In ref. 89 particle characterization of suspended nanoparticles using iSCAT (A) was demonstrated through quantitative analysis of the scattered light in combination with particle tracking (B). The iSCAT contrast was shown to be proportional to particle volume for small particles (C, upper row), as anticipated from section 4, and the deviation from this scaling was used to estimate the internal refractive index of extracellular vesicles (B, lower row). (D) By utilizing the interference between a nanochannel and particles residing within the nanochannel, in ref. 16, characterization of polarizability (proportional to mass) as well as the hydrodynamic radius of individual biomolecules was demonstrated. Figure reprinted with permission under the CC-BY 4.0 license.

Interferometric methods enable accurate particle characterization both on a surface^27^ and when in solution.^16,89^ In ref. 89, signal quantification in combination with particle tracking using iSCAT (Fig. 13A) was used to determine the size and refractive index of suspended nanoparticles (Fig. 13B and C). Moreover, by analyzing the particle-size scaling, they could obtain structural information about suspended liposomes. To investigate even smaller suspended particles, in ref. 16, they used the relative scattering between a nanochannel and the particle to characterize the suspended size and mass of individual biomolecules possible (Fig. 13D).

**Fig. 14:**
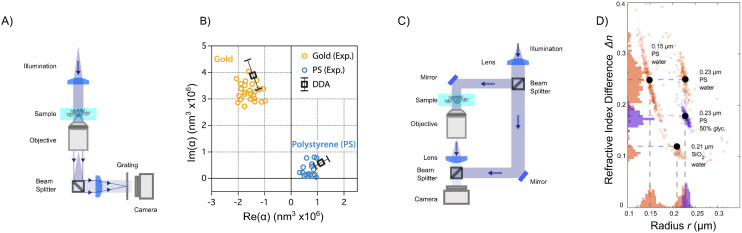
Particle characterization using holographic imaging. (A) and (B) Using quadriwave lateral shearing interferometry (QLSI), in ref. 67 it was demonstrated that gold and dielectric nanoparticles can be distinguished based on their complex-valued polarizabilities. (C) and (D) In ref. 19 it was demonstrated that deep learning enhanced analysis of particle scattering patterns recorded in off-axis holography is capable of quantifying the size and refractive index of suspended subwavelength particles. Figure reprinted with permission under the CC-BY 4.0 license.

A fourth measurement consideration is whether more detailed material information is needed in the case of heterogeneous samples. Holographic imaging provides the most rich optical signal that can, in turn, be used for the detailed characterization of nanoparticles. For instance, the complex-valued scattered field contains information about both the real and imaginary parts of the particle polarizability and refractive index (Fig. 14A and B).^21,67^ In ref. 67, this was used to distinguish between gold nanoparticles and polystyrene particles directly from the optical signal. Similarly, in ref. 14, the sign of the phase signal was used to differentiate between nanobubbles and dielectric particles in the same sample. Furthermore, in ref. 19, the complex-valued signal was used to determine the size and refractive index of nanobeads and fractal aggregates directly from the scattered light, without invoking particle tracking (Fig. 14C and D).^19^ Thus, the complex-valued optical field in holographic provides more detailed information about the particle material of the measured particles than darkfield and interferometric imaging.

One major drawback of holographic microscopy techniques compared to other optical techniques has been its detection limit. However, it was recently shown that by combining evanescent field imaging with an external reference, it is possible to measure the optical field from single proteins with mass below 100 kDa when binding to a surface.^28^

This leads to another critical consideration when deciding on a measurement technique, namely particle size. The lowest reported detection limit is 9 kDa biomolecules measured using iSCAT (corresponding to a diameter of approximately 3 nm).^91^ As a comparison, the reported detection limit for darkfield microscopy is around 5 nm diameter for non-metallic particles,^12^ iSCAT for suspended nanoparticles has a reported detection limit of around 40 nm,^15^ and holographic microscopy measurements for suspended nanoparticles has a reported detection limit of around 70 nm.^21^ In addition to the detection limit, as described in section 5.2, transmission methods and non-transmission methods have different relationships between particle signal and polarizability. For particle mass determination, it is beneficial to use a measurement geometry for which the form factor contribution to the scattered light can be neglected. In practice, this implies that the illumination angle ideally should be related to the typical radius *R* of scatterers in the sample as sin(*θ*_ill_/2) < (2*kR*)^−1^.

Generally speaking, the more parameters that can be quantified at the single particle level, the more likely it is that the particles of interest can be distinguished from other particles in the sample. For instance, if the particles of interest are the strongest scattering particles, then any technique that permits quantifying polarizability is sufficient. If the particles scatter light to a similar extent but have a different material composition, then particle refractive index may be the relevant parameter to characterize. Finally, if the particles of interest differ from other particles in how the mass is distributed within the particle, then some technique capable of resolving the form factor is preferable. If these physical parameters are insufficient for distinguishing the particles of interest from other particles in the sample, it is also possible to augment elastic scattering microscopy techniques with fluorescent imaging to achieve better specificity.

## 7 Conclusions and future opportunities

In this tutorial, we have given an overview of the particle information from darkfield, interferometric scattering, and holographic microscopy measurements. Over the past decades, the single particle detection limit has significantly improved for all different optical imaging methods. Looking ahead, there are still opportunities and challenges for scattering-based microscopy characterization beyond detection. This includes multiparametric characterization of individual particles in terms of particle material, size, and shape, particularly for particles in complex environments such as inside cells.

### 7.1 Optical fingerprinting

A fundamental limitation in scattering-based particle characterization is that the detection events are nonspecific, as mentioned in the previous section. A challenge in the field of scattering-based nanoparticle characterization is to identify optical fingerprints, which enable distinguishing and characterizing subpopulations in heterogeneous samples without introducing labels.

One such fingerprinting feature, the integrated scattering amplitude, has been discussed at length in this tutorial. This feature is proportional to particle polarizability and can be used to distinguish subpopulations. Another experimentally measurable feature is the hydrodynamic radius, estimated through particle tracking.^13,15,16^

By combining the information-rich microscopy images containing scattering patterns of nanoparticles with deep learning-enhanced analysis techniques that go beyond quantifying the integrated scattering signal, we anticipate that more examples of fingerprinting features will be added to this list, enabling more precise population discrimination and characterization. To give a few examples:

• In principle, holographic imaging techniques can quantify the scattering form factor across all scattering angles collected by the objective. Utilizing this, it is possible to discriminate particle subpopulations based on their morphology.

• In biological systems, many processes are driven by weak interactions. In a elastic scattering microscope, such interactions will manifest themselves as temporal fluctuations in particle properties.^19^ Such temporal fluctuations can also be used as a fingerprinting feature.

• The signal amplitude in interferometric scattering microscopy images is, just as the complex-valued optical field measured in holographic microscopy, related to the form factor evaluated over the scattering angles captured by the objective. This information can likely be decoded using deep learning enhanced analysis techniques, enabling precise sizing of very small objects.

• Shape information of anisotropic particles is also encoded in the scattering patterns in a microscopy image. For anisotropic nanoparticles, the scattering is different compared to spherical particles, as highlighted in context of eqn (33)–(35). For example, for suspended anisotropic particles, the scattered light reaching the camera will temporally fluctuate as the nanoparticle undergoes rotational diffusion. If the exposure time is much shorter than the characteristic time of rotational diffusion, these fluctuations can be resolved in measurement and be related to particle anisotropy.^73^ Thus, optical characterization of anisotropic particles has some additional opportunities and challenges compared to isotropic particles, where the extent of the signal difference due to anisotropic particles also depends on measurement geometry and which microscopy method that is used.

• Finally, the information about the scattering form factor within an image captured in a elastic scattering microscope is limited by the scattering angles collected by the objective. Thus, different scattering approaches carry complementary information about the scattering form factor. By combining measurement modalities, it is possible to obtain a more complete mapping of the scattering form factor of individual nanoparticles. This was recently demonstrated by combining holographic imaging and iSCAT to quantify particle size in unknown sample media,^23^ and we anticipate that the same idea will be applied using other combinations of scattering techniques as well.

The primary obstacles to achieving such fingerprinting lie in the noise level of interferometric systems, obscuring parts of the scattering signal, and an imperfect characterization of the pupil function *P*(*θ*). In the treatment presented in this tutorial, this has been assumed to be perfectly characterized. In practice, this function is affected by aberrations in the optical system and is difficult to characterize perfectly. Furthermore, in this tutorial, all particles have been assumed to be located directly above the central line of the objective. In a real experiment, the position of the particle with respect to the center of the objective will affect the scattering angles that reach the objective. Thus, the image of a particle will be slightly affected by its position.^92^ These effects were characterized and accounted for in ref. 19 using holographic microscopy, where calibration particles were used to obtain information about the occurring point spread function, but performing such characterization for other elastic scattering microscopy geometries has not been demonstrated. Nonetheless, considering the fast improvement of the detection limits of scattering-based microscopy^27,28^ we anticipate that deep learning enhanced analysis of microscopy images will enable precise nanoparticle characterization over a wide range of particle sizes and shapes.

### 7.2 Characterizing particles in complex environments

Most single particle characterization methods operate in known environments at a controlled particle concentration. Operating in unknown and crowded environments adds several challenges, including the unknown viscosity hindering estimation of the hydrodynamic radius, unknown media refractive index hindering particle mass measurements, and overlapping scattering patterns may hinder single particle tracking.

For example, surrounding biomolecules may bind to the particles^93^ as well as affect the surrounding refractive index.^26^ Since the optical scattering depends on the relative difference in refractive index between the particle and the surrounding media, an unknown surrounding refractive index limits the ability of quantifying the refractive index of the measured particle. Moreover, in the case of biological nanoparticles such as liposomes and extracellular vesicles, particle media may also enter into the particle, which affects the effective refractive index of the particle.^94^ Other media properties such as pH may for example influence the water content inside the particles.^95^ Thus, it is critical to consider how the particle media may affect the measured signal and the subsequent estimates of particle properties.

All-optical fingerprinting of nanoparticles in complex environments therefore need to alleviate the need for known viscosity and surrounding media. For highly scattering particles, deep learning approaches can be used to identify and characterize the particles inside cells.^85^ For example, using deep learning-assisted image analysis combined with quantitative field imaging it is possible to estimate particle size and relative refractive index in unknown media.^19,23^ When using scattering amplitude ratios from measurements at different scattering angles rather than from detailed analysis of the scattering pattern in a single microscopy image, as in ref. 23, particle size can be estimated in unknown sample environments directly from the optical signal without the need of detailed information about the optical transfer function of the microscope. Thus, measurement strategies along those lines have the potential to be generalized for particle characterization in complex sample environments where media refractive index and viscosity are unknown.

For less strongly scattering particles, scattering from nearby particles in crowded environments such as inside cells may hinder the ability to identify single particles. One way to overcome this limitation is to use confocal methods such as confocal iSCAT.^96,97^ During confocal microscopy measurements only a small volume of the sample is illuminated at a time, where the depth selectivity of confocal microscopy significantly reduces the background scattering from other particles, allowing for example tracking of individual viruses on a cell.^96^ However, since the optical scattering signal depends on the relative refractive index difference, it remains a challenge to relate the scattering signal to particle properties such as mass and size. Although it might be challenging to combine confocal iSCAT with measurement concepts such measuring the scattering at different scattering angles to obtain particle size, other scattering ratio such as simultaneous measurements at different wavelengths have a similar theoretical potential to be used to estimate particle size. Thus, the combination of confocal elastic scattering microscopy and deep learning image analysis has the potential to significantly extend the quantitative possibilities of label-free optical particle characterization.

## Data availability

The code for detecting and characterizing nanoparticles can be found at https://github.com/softmatterlab/OpticalCharacterizationNanoparticles.

## Conflicts of interest

DM and EO owns shares in a company that holds IP for quantification of particles in off-axis holographic imaging (HOLTRA). FH owns shares in the company Nanolyze, developing optical waveguide chips for surface sensitive scattering. DM and GV own shares in the company IFLAI, providing AI-based tools to analyze microscopy data.

## Supplementary Material

NR-017-D4NR03860F-s001
