# Supplementary material for: Optical label-free microscopy characterization of dielectric nanoparticles
Source: Nanoscale. 2025 Feb 25;17(14):8336–62. doi: 10.1039/d4nr03860f (PMC11904879; doi:10.1039/d4nr03860f)
Supplement: NR-017-D4NR03860F-s001 [file NR-017-D4NR03860F-s001.pdf]

Supplementary Information (SI) for Nanoscale.
This journal is © The Royal Society of Chemistry 2025

Supplementary Information to: Optical Label-Free Microscopy Characterization of Dielectric Nanoparticles

Berenice García Rodríguez[1,*], Erik Olsén[2,*], Fredrik Skärberg[1,*], Giovanni Volpe[1], Fredrik Höök[2], and Daniel Sundås Midtvedt[1]

[1]Department of Physics, University of Gothenburg, Gothenburg, Sweden
[2]Department of Physics, Chalmers University of Technology, Gothenburg, Sweden
[*]These authors contributed equally to this work, and are listed in alphabetical order.

February 11, 2025

1 Light scattering from a nanoparticle illuminated by linearly polarized light

Consider a spherical nanoparticle illuminated by an optical field polarized in the x-direction and propagating along the z-direction, such that the field is written

$$\vec{E} = E_x e^{ikz}\hat{x}. \tag{1}$$

The scattering of light from the nanoparticle is described by [1]

$$\vec{E}_{\text{sca}}(r,\theta,\phi) = \frac{e^{ik(r-z)}}{ikr}\left(S_1(\theta)\sin\phi E_x\hat{e}_\perp + S_2(\theta)\cos\phi E_x\hat{e}_\parallel\right), \tag{2}$$

where S_1, S_2 are called the scattering matrix elements, and r is the distance from the center of the scatterer. Further, $\hat{e}_\parallel$ and $\hat{e}_\perp$ are unit vectors parallel and perpendicular to the scattering plane, defined by the scattering direction $\hat{e}_r$ and the direction of propagation of the illuminating light $\hat{z}$. Parametrizing the scattering direction by the scattering angle θ, measured relative to the propagation direction, and the azimuthal angle ϕ, measured relative to the polarization direction (x) of the illuminating field, the unit vectors are given by

$$\begin{aligned}\hat{e}_\parallel &= -\cos\theta\cos\phi\hat{x} - \cos\theta\sin\phi\hat{y} + \sin\theta\hat{z}\\ \hat{e}_\perp &= -\sin\phi\hat{x} + \cos\phi\hat{y}.\end{aligned} \tag{3}$$

The scattering matrix elements for a weakly scattering nanoparticle are given by

$$\begin{aligned}S_1 &= \frac{ik^3\alpha}{2\pi}f(\theta;R)\\ S_2 &= \frac{ik^3\alpha}{2\pi}\cos\theta f(\theta;R)\end{aligned} \tag{4}$$

where $f(\theta;R)$ is the optical form factor of the particle and α is the polarizability. Utilizing these expressions one finds

$$\vec{E}_{\text{sca}}(r,\theta,\phi) = \frac{e^{ik(r-z)}}{kr}\frac{k^3\alpha}{2\pi}f(\theta;R)\left[\left(\cos^2\theta\cos^2\phi + \sin^2\phi\right)\hat{x} + (1-\cos^2\theta)\cos\phi\sin\phi\hat{y} + \cos\theta\sin\theta\cos\phi\hat{z}\right] \tag{5}$$

This describes an optical field with plane wave decomposition

$$\hat{E}_{\text{sca,x}} = \frac{k\alpha}{2\pi} f(\theta; R) \left(\cos^2\theta \cos^2\phi + \sin^2\phi\right) \tag{6}$$

$$\hat{E}_{\text{sca,y}} = \frac{k\alpha}{2\pi} f(\theta; R)(1 - \cos^2\theta) \cos\phi \sin\phi \tag{7}$$

$$\hat{E}_{\text{sca,z}} = \frac{k\alpha}{2\pi} f(\theta; R) \cos\theta \sin\theta \cos\phi, \tag{8}$$

which, for $f = 1$ reduces to the expression found in the main text.

The polarization will contribute to the measurements differently depending on the measurement geometry. In the case of interferometric imaging, only the x-components will contribute to the interference signal. According to Box 8 in the main text, the only contribution to the integrated signal comes from $\theta = \theta_{\text{ill}}$, for which $\cos^2\theta = 1$, and thus the polarization factor $\cos^2\theta\cos^2\phi + \sin^2\phi \equiv 1$. Thus, the polarization does not affect interferometric measurements of the integrated signal.

In the case of darkfield measurements, the situation is slightly different. In the case of $\theta_{\text{ill}} = 0$ or $\theta_{\text{ill}} = \pi$, the z-axis is an axis of symmetry, simplifying the problem. In this case, the integrated intensity at the camera plane is given by

$$\int I_{\text{cam}} d\vec{x} = \frac{k^4\alpha^2|E_x|^2}{2\pi} \int (|E_{\text{sca,x}}|^2 + |E_{\text{sca,y}}|^2 + |E_{\text{sca,z}}|^2) \sin\theta \cos\theta d\theta d\phi. \tag{9}$$

This can be simplified to

$$\int I_{\text{cam}} d\vec{x} = k^4\alpha^2|E_x|^2 \int d\theta \sin\theta \cos\theta f(\theta)^2(\cos^2\theta + 1), \tag{10}$$

which is identical to the expression derived in the main text using scalar diffraction theory. Thus, scalar diffraction theory produces exactly correct results for the integrated signal of spherically symmetric nanoparticles.

The case of illumination angle $\theta = \pi/2$ is analogous, but with the caveat that the angles ϕ and θ are no longer independent. Specifically, one can write [2]

$$\phi = \arcsin\left(\frac{\sin\theta_{\max}}{\sin\theta}\right), \tag{11}$$

such that the integrated intensity becomes

$$\int I_{\text{cam}} d\vec{x} = \frac{k^4\alpha^2|E_x|^2}{4\pi^2} \int_{\pi/2-\theta_{\max}}^{\pi/2+\theta_{\max}} d\theta \int_{\pi/2-\phi(\theta)}^{\pi/2+\phi(\theta)} d\phi \sin\theta f(\theta)^2 \left(\cos^2\theta + 1\right). \tag{12}$$

References

[1] C. F. Bohren and D. R. Huffman, *Absorption and scattering of light by small particles.* John Wiley & Sons, 2008.

[2] J. Fattaccioli, J. Baudry, J.-D. Émerard, E. Bertrand, C. Goubault, N. Henry, and J. Bibette, "Size and fluorescence measurements of individual droplets by flow cytometry," *Soft Matter*, vol. 5, no. 11, pp. 2232–2238, 2009.
